# Synthesis and Catalytic Performance of Bimetallic Oxide-Derived CuO–ZnO Electrocatalysts for CO_2_ Reduction

**DOI:** 10.1021/acscatal.4c01575

**Published:** 2024-07-02

**Authors:** Matt L. J. Peerlings, Kai Han, Alessandro Longo, Kristiaan H. Helfferich, Mahnaz Ghiasi, Petra E. de Jongh, Peter Ngene

**Affiliations:** † Materials Chemistry and Catalysis, Debye Institute for Nanomaterials Science, Utrecht University, 3584 CG Utrecht, The Netherlands; ‡ European Synchrotron Radiation Facility (ESRF), 71, Avenue des Martyrs, Grenoble F-38000, France; § Istituto per lo Studio dei Materiali Nanostrutturati (ISMN)-CNR, UOS Palermo, via Ugo La Malfa 153, Palermo 90146, Italy

**Keywords:** electrochemical CO_2_ reduction, bimetallic Cu−Zn catalyst, phase separation, oxidation state, electronic modification, stability

## Abstract

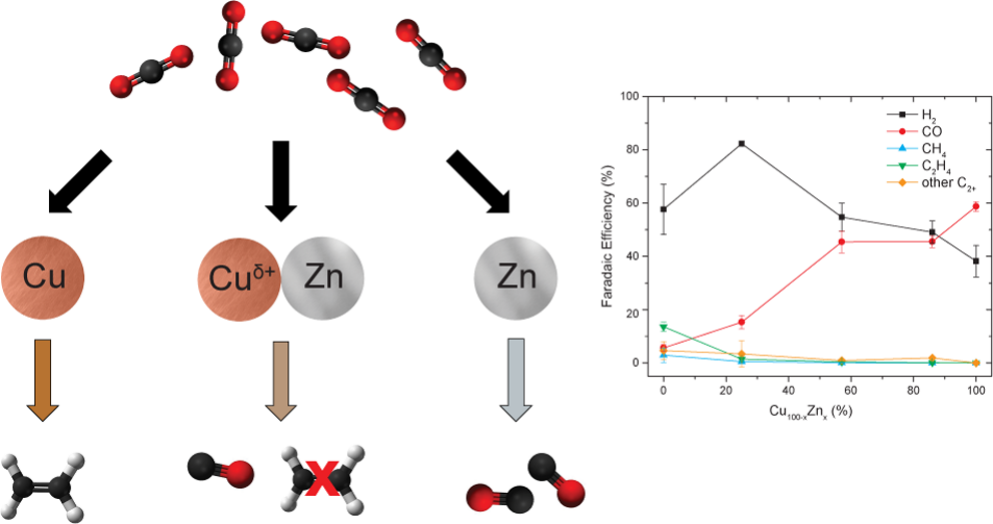

Steering the selectivity of electrocatalysts toward the desired product is crucial in the electrochemical reduction of CO_2_. A promising approach is the electronic modification of the catalyst’s active phase. In this work, we report on the electronic modification effects on CuO–ZnO-derived electrocatalysts synthesized via hydrothermal synthesis. Although the synthesis method yields spatially separated ZnO nanorods and distinct CuO particles, strong restructuring and intimate atomic mixing occur under the reaction conditions. This leads to interactions that have a profound effect on the catalytic performance. Specifically, all of the bimetallic electrodes outperformed the monometallic ones (ZnO and CuO) in terms of activity for CO production. Surprisingly, on the other hand, the presence of ZnO suppresses the formation of ethylene on Cu, while the presence of Cu improves CO production of ZnO. *In situ* X-ray absorption spectroscopy studies revealed that this catalytic effect is due to enhanced reducibility of ZnO by Cu and stabilization of cationic Cu species by the intimate contact with partially reduced ZnO. This suppresses ethylene formation while favoring the production of H_2_ and CO on Cu. These results show that using mixed metal oxides with different reducibilities is a promising approach to alter the electronic properties of electrocatalysts (via stabilization of cationic species), thereby tuning the electrocatalytic CO_2_ reduction reaction performance.

## Introduction

A promising strategy to close the carbon cycle is the electrochemical CO_2_ reduction reaction (CO_2_RR) to chemicals and fuels using renewable electricity.^1,2^ This is necessary to meet the Paris accord on climate change, which aims to keep the global temperature rise well below 2 °C above preindustrial levels.^3^ To date, a wide variety of electrochemical CO_2_ reduction products can be generated depending on the catalyst material used. Different metals are reported to be active CO_2_RR electrocatalysts, and they have been categorized based on their main products. These products include CO (on Au, Ag, Zn), formate (on Sn, Pb, Bi), and hydrocarbons (on Cu).^4^ In the latter case, CO is identified as the key intermediate for further hydrocarbon production.^5^ Furthermore, carbon monoxide (CO) is widely used as an important feedstock for the production of methanol and other valuable chemicals. Therefore, the electrochemical conversion of CO_2_ to CO is currently being scaled up for industrial applications using Ag-based electrodes.^6^ However, the process still suffers from selectivity issues, mainly due to the competing hydrogen evolution reaction (HER). Furthermore, improvements in terms of current density, overpotential, and process stability are essential for scaling up this process.^7^

Although Ag and Au have shown promising CO_2_RR performance, using these precious metals might not be the best option for making affordable electrolyzers. Instead, metal–nitrogen-doped carbon materials might be more suitable due to their use of earth-abundant materials, low CO_2_RR to CO overpotentials, and high current densities.^8,9^ A different cost-affordable option is the use of Zn because it is an earth-abundant and cheap metal.^4^ Despite good CO_2_RR to CO selectivity, Zn-based CO_2_RR electrodes are however less attractive due to high required overpotentials for selective CO production, low catalyst activity, and stability issues.^10^ Therefore, it is important that approaches are developed to increase their catalytic performance in order to make them a viable alternative to other catalyst materials. On the other hand, although Cu makes CO and other hydrocarbon products at moderate potentials, it suffers from poor selectivity toward any one of the desired products.^11,12^

Thus, far, several approaches have been made to tune the binding strength of reaction intermediates and, hence, the electrocatalytic performance of Zn- and Cu-based electrodes. Two of these approaches include tailoring the catalyst structure and using oxide-derived catalysts.^13^ For instance, by manipulating the structure of the Zn catalyst into a hierarchical hexagonal shape, a high CO_2_ to CO Faradaic Efficiency (FE) of 95% (at −1.05 V in 0.5 M KCl) could be achieved, as well as a high stability (5% activity loss in 30 h).^14^ A study by Luo et al.^10^ found that when using oxide-derived Zn, regardless of the initial structure, the ZnO undergoes severe restructuring to porous structures composed of hexagonal Zn crystals. These oxide-derived reconstructed catalysts displayed a high CO FE above 90% and stability of more than 18 h, as well as a similar intrinsic CO activity after normalization for their electrochemical surface area (ECSA).^10^ In a different study, Geng et al. showed that the introduction of oxygen vacancies via a H_2_ plasma treatment could increase both the CO FE from 44 to 83%, as well as the CO partial current density by a factor of 5.1, compared to starting from pristine ZnO nanosheets.^15^ More recently, we studied nanorod-shaped ZnO catalysts and demonstrated that the morphology of the resulting Zn catalyst could be tuned via different reduction procedures. It was found that the final structure of the active Zn catalysts significantly affected both the activity and product selectivity for CO_2_ reduction.^16^

A different approach to improve the performance of CO_2_RR electrocatalysts is to combine two different metals.^17^ For example, Feng et al. demonstrated the principle of tandem catalysis, in which Zn is added to Cu in order to increase the *CO coverage on Cu and hence promote the formation of ethylene.^18^ Interestingly, however, Ren et al. found that adding Zn to Cu shifts the selectivity to ethanol instead of ethylene formation.^19^ Likely, catalyst restructuring and oxidation state play roles in these seemingly contradicting results. In an effort to disentangle these contributions, da Silva et al. studied copper-rich bimetallic CuZn-based catalysts. They showed that the catalyst morphology had the strongest effect on the selectivity to C_2+_ products, although other factors such as atomic composition, oxidation state, and surface roughness were also found to play a role in the overall catalyst performance.^20^ However, C_2+_ products are not always formed on CuZn-based catalysts, as in the work of Jeon et al.^21^ They studied well-defined Cu–Zn nanoparticles of ca. 5 nm with a wide range of atomic compositions. The main products formed were CH_4_, H_2_, and CO, depending on the atomic composition. Increasing the Zn content decreased the CH_4_ and increased syngas (CO/H_2_) selectivity, corresponding to the formation of CuZn alloys instead of nonfully reduced Cu-ZnO.^21^ Consequently, the catalysts also exhibit time-dependent selectivity because at longer reaction times, more cationic Zn species are reduced and CuZn alloy is formed, favoring H_2_ over CH_4_ production.^21,22^ However, the effect of restructuring on time-dependent selectivity was not studied in detail. Interesting in this regard is the study by Wan et al.,^23^ which demonstrates bimetallic Cu–Zn catalysts selective toward CO. The CO activity and stability were found to be higher in the case of a phase-separated sample than a core–shell one due to zinc enrichment at the catalyst surface in the latter case.

These studies demonstrate that combining oxide-derived Zn and Cu is a promising approach to tune the catalyst selectivity. Furthermore, the catalyst structure plays an important role, although much is still unclear about the impact of restructuring on the electrocatalytic performance. In this study, we focus on the catalytic performance of oxide-derived CuO–ZnO catalysts prepared via a hydrothermal synthesis method. Remarkably, although the synthesis method led to structurally distinct and spatially separated CuO and ZnO, the oxides undergo massive restructuring and intermixing during CO_2_RR. The resulting close contact between copper and zinc affects their reducibility and thereby leads to a significant change in the product selectivity due to the stabilization of cationic Cu species by partially reduced ZnO, and enhanced reducibility of the ZnO by Cu. Therefore, our work reveals that the stabilization of cationic metal species by the presence of another metal oxide with less reducibility is a promising approach to tune the catalytic performance of CO_2_ reduction electrocatalysts.

## Experimental Section

### Cu_1–*x*_Zn_*x*_O Preparation

The bimetallic Cu_1–*x*_Zn_*x*_O catalysts were grown on carbon paper (TGP-H-060) using a modified hydrothermal method based on previous work for ZnO.^16,24^ Typically, a piece of carbon paper disc (*d* = 2.5 cm) was immersed into an aqueous solution containing 0.05 M M(NO_3_)_2_ (M = Zn or Cu) and 0.05 M hexamethylenetetramine (HMT). The solution was transferred to a Teflon liner and sealed in an autoclave reactor. The autoclaves were subsequently kept at 100 °C for 5 h. Afterward, the carbon paper was washed with Milli-Q water and dried under ambient conditions. By change of the ratio of copper to zinc nitrate, catalysts with different atomic compositions were prepared.

### Structural Characterization

XRD measurements were performed with a Bruker D2 Phaser, equipped with a Co Kα X-ray source (λ = 1.79026 Å). The XRD measurements were conducted at a 2θ range from 35 to 80° using 1 s as the integration time. SEM-EDX micrographs and elemental maps were made on a Zeiss EVO 15 instrument equipped with a secondary electron detector, operating at 400 pA and 10.0 kV for imaging and at 20.0 kV for elemental maps. High-resolution SEM images were made on a Zeiss Gemini instrument equipped with an InLens detector and operated at 500 pA and 10.0 kV. Bright field transmission electron microscopy (BF-TEM) and high angle annular dark field scanning transmission electron microscopy (HAADF-STEM) were performed on the Talos F200x operated at 200 kV. Energy dispersive X-ray spectroscopy (EDX) was employed to identify and map the present elements. Samples were prepared by drop casting onto a gold-coated carbon grid after the dispersion of the catalysts by ultrasonication in ethanol. Inductively coupled plasma (ICP) analysis was carried out using a PerkinElmer AAS Model Analyst 200 at MIKROLAB (Mikroanalytisches Labor Kolbe, c/o Fraunhofer Institut UMSICHT, Germany).

### Electrochemical Measurements

The electrochemical experiments were performed in an H-type cell (Figure S1) using a PARSTAT MC potentiostat. In the cell, both the cathode and anode compartments are filled with 15 mL of 0.1 M KHCO_3_ electrolyte solution, separated by a Fumasep membrane (FAA-3-PK-130, Fumatech BWT, GmbH). The cathode and anode compartments were flushed for 30 min before and during the experiments using 20 mL/min CO_2_ and Ar, respectively. In the cell configuration, the carbon paper-supported catalyst was pressed onto a piece of glassy carbon current collector. A Ag/AgCl (3 M KCl) reference electrode and commercial iridium oxide-based counter electrode (Dioxide Materials) were used. The exposed geometric area of both the working and counter electrodes is 3.8 cm^2^.

All electrodes were tested at five increasingly cathodic potentials for two cycles, of which the second cycle was used to determine the selectivity. Subsequently, the electrodes were kept at the maximum cathodic potential for long-term testing. All potential values were converted to potentials with respect to the reversible hydrogen electrode potential (RHE) and *iR*-corrected using a typical resistance of around 28 Ω in 0.1 M KHCO_3_ solution as determined from electrochemical impedance spectroscopy (EIS) measurements. Typically, 80% *iR* correction was applied during each measurement, and the remainder was corrected afterward. All results shown are the average of three independent measurements. Error bars display the standard deviation.

### Gas Product Detection

Gaseous products were analyzed using a Global Analysis Solutions Microcompact GC 4.0. The GC system was equipped with 3 detector channels (2 FID and 1 TCD). The first channel has a Rt-Q Bond (10 m × 0.32 mm, Agilent) packed column and an FID detector for the detection of CH_4_, C_2_H_4_, and C_2_H_6_; the second channel has a Molecular Sieve 5A (10 m × 0.53 mm, Restek) packed column that separates small gaseous molecules such as CO and CH_4_. This channel has an FID detector with a methanizer to increase the detection sensitivity of CO. The third channel has a Carboxen 1010 (8 m × 0.32 mm, Agilent) packed column which separates H_2_ and CO_2_ with a TCD. High purity nitrogen (N_2_, 99.999%) was used as carrier gas.

### Liquid Product Detection

Products in the liquid phase were analyzed by taking 1 mL of electrolyte samples after each potential step and replacing this with fresh 0.1 M KHCO_3_. 100 μL amount of internal standard solution containing 10 mM DMSO and 50 mM phenol in D_2_O was added to each 500 μL electrolyte sample and analyzed using ^1^H NMR with solvent suppression on a 400 MHz VNMRS-400 Varian NMR. Products were quantified by comparing the product and internal standard peak areas and correcting for sampling volume.

### *In Situ* XAS Measurements

X-ray absorption measurements were performed at the LISA beamline (BM 08) of the European Synchrotron Radiation Facility (ESRF) in Grenoble, France. For the *operando* XAS measurements, a homemade XAS electrochemical cell was used (Figure S2). A CO_2_-saturated 0.1 M KHCO_3_ electrolyte was flown through the cell with a flow rate of 3 mL/min using a peristaltic pump. The catalysts were used as the working electrode, a platinum wire as counter electrode, and a Ag/AgCl 3 M KCl electrode as reference electrode. A BioLogic SP-50 potentiostat was used for all *in situ* XAS experiments. No *iR* correction was applied.

All *in situ* XAS experiments were performed in fluorescence mode with an angle of 45° between the incoming X-rays and the sample. Spectra were recorded at the Cu K-edge (8979 eV) and Zn K-edge (9659 eV), respectively. The time to acquire a spectrum was 80 min for recording both the Cu and Zn spectra at each potential. Reference spectra of ZnO, Zn, CuO, and Cu were acquired in transmission mode. Both Athena and GNXAS data processing software were used for the analysis of data. The spectra (two scans per sample) were energy-calibrated, averaged, and further analyzed using GNXAS.^25^ In this approach, the local atomic arrangement around the absorbing atom is decomposed into model atomic configurations containing 2, ..., *n* atoms. The theoretical EXAFS signal *c*(*k*) is given by the sum of the *n*-body contributions *g*^2^, *g*^3^, ..., *g*^*n*^ which takes into account all of the possible single and multiple scattering (MS) paths between the *n* atoms. The modeling of *c*(*k*) to the experimental EXAFS signal allowed us to refine the relevant structural parameters of the different coordination shells. The quality of the model is also checked by comparison of the experimental EXAFS signal Fourier transform (FT) with the FT of the calculated *c*(*k*) function. The coordination numbers and the global fit parameters that were allowed to vary during the fitting procedure were the following: the distance *R* (Å), Debye–Waller factor (*s*^2^), and the angles of the *g*^*n*^ contributions. The edge energy *E*_0_ was fixed at the Cu K-edge (8979 eV) and Zn K-edge (9659 eV) corresponding values.

## Results and Discussion

### Structural Characterization of the Cu_1–*x*_Zn_*x*_O Catalysts

To assess the elemental composition of the Cu_1–*x*_Zn_*x*_O electrodes prepared using hydrothermal synthesis, inductively coupled plasma (ICP) measurements were carried out. The results are summarized in Table 1. From these results, it follows that a series of electrodes with systematically varying Cu:Zn ratios have been prepared. Because some copper precipitation was already observed upon mixing the reagents, the obtained Cu:Zn ratio is lower than the precursor ratio in solution, and the total catalyst loading decreases slightly with addition of a higher relative amount of copper.

**Table 1:** ICP Results of the Electrodes^a^

	Cu:Zn atom %		
sample	in solution	on electrode	metal loading on C paper (wt %)
ZnO	0	0	2.9
Cu_0.14_Zn_0.86_O	50	14	1.8
Cu_0.43_Zn_0.57_O	67	43	1.5
Cu_0.75_Zn_0.25_O	75	75	1.1
CuO	100	100	0.8

a Indicating the atom % of Cu with respect to Zn in the hydrothermal synthesis precursor solution and on the fabricated electrodes; additionally, the metal oxide weight loadings on the electrodes are reported.

**Figure 1:**
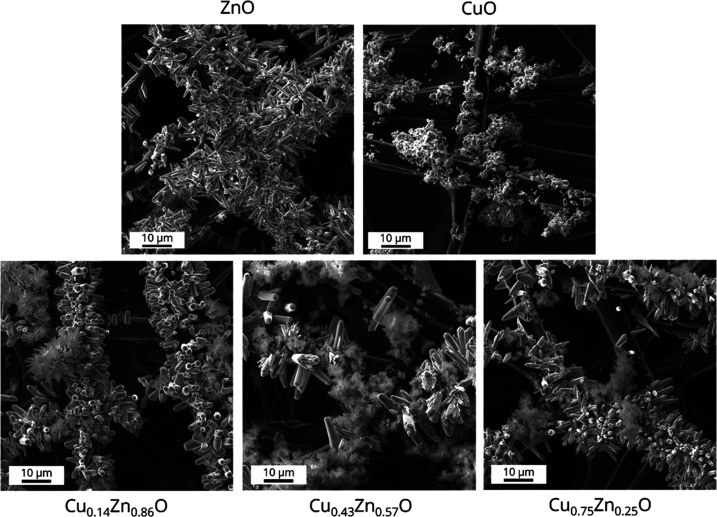
SEM images of freshly prepared Cu_1–*x*_Zn_*x*_O electrodes. The ZnO particles have a rod-like shape, whereas CuO particles are more amorphous.

**Figure 2:**
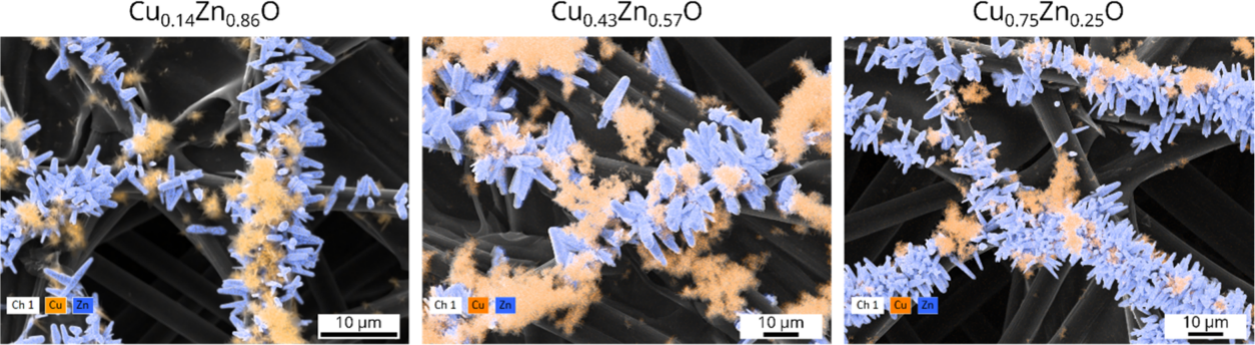
SEM-EDX elemental maps of freshly prepared Cu_1–*x*_Zn_*x*_O-based electrodes showing the Cu (orange) and Zn (blue) elemental distributions. Zn is located in the rod-shaped particles, whereas Cu is mainly present in the more amorphous ones.

To further evaluate the structure of the Cu_1–*x*_Zn_*x*_O catalysts, scanning electron microscopy (SEM) images were made as shown in Figures 1 and S12. For ZnO particles, a rod-like shape is observed, in line with previous results.^16,24^ In contrast, CuO is present in a less well-defined shape. These results agree with the XRD patterns in Figure S3, which show high crystallinity and a preferential growth direction for ZnO, in contrast to weak diffraction lines for CuO. In the bimetallic samples, both rod-shaped particles and less faceted particles are observed. In agreement with the findings for the monometallic electrodes, the elemental maps confirm that the rod-shaped particles contain mainly zinc, whereas the less faceted particles contain mostly copper (Figure 2). Furthermore, the images indicate that copper and zinc are not mixed homogeneously, but rather they are present in separate structures. Indeed, it is known that bulk CuO and ZnO do not form mixed phases,^18^ unlike their metallic counterparts which can coexist in different compositions.^20^

### Electrochemical Performance of the Cu_1–*x*_Zn_*x*_O Catalysts

**Figure 3:**
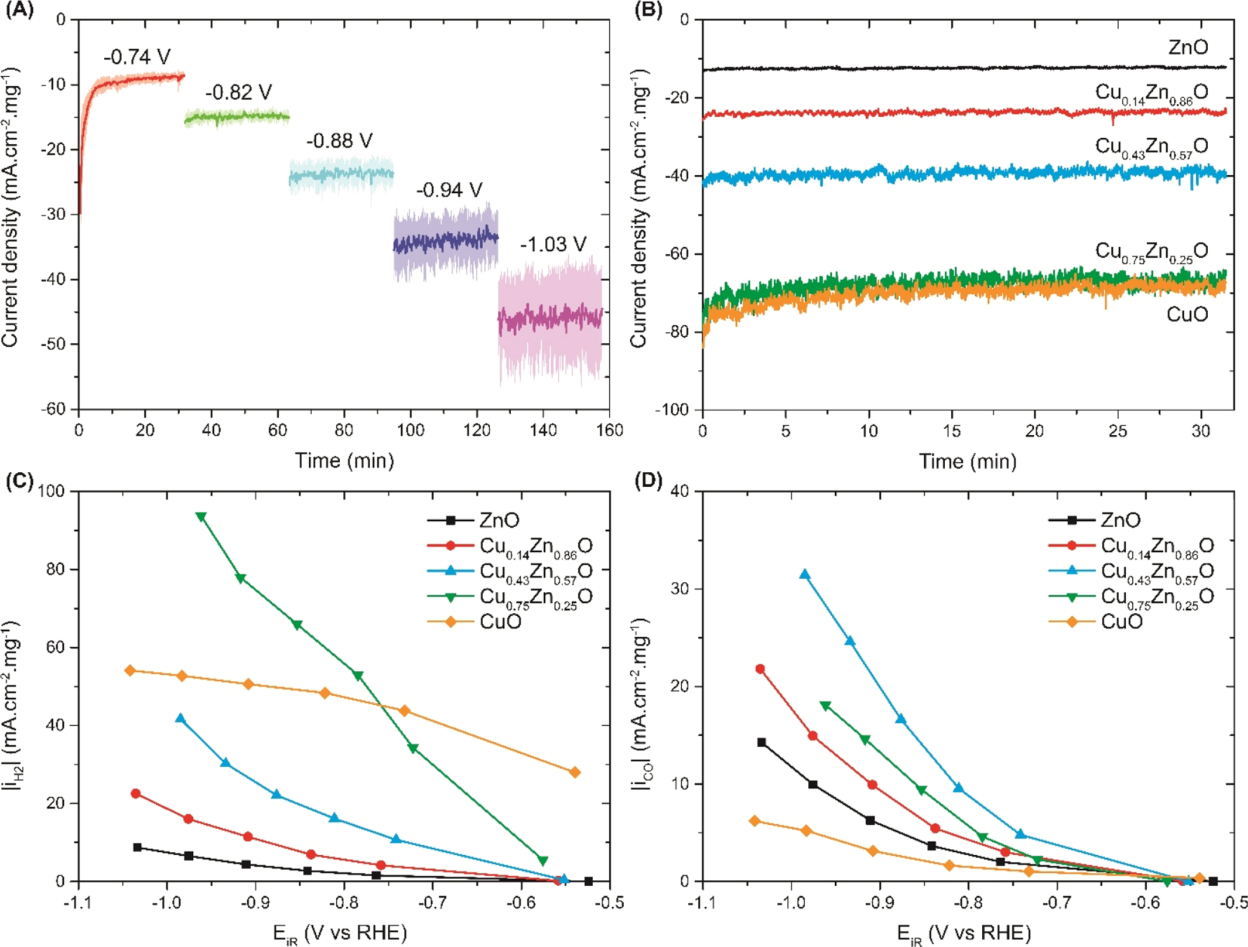
Electrochemical evaluation of Cu_1–*x*_Zn_*x*_O catalysts. (A) Chronoamperometry data of Cu_0.14_Zn_0.86_O at five consecutive applied potentials. The indicated lines are an average of three independent measurements, with the shaded areas indicating the standard deviation. (B) Current density of all electrodes at −0.9 V vs RHE. Partial current densities to H_2_ (C) and CO (D) of all electrodes vs *iR*-corrected potential. All currents are normalized by geometric surface area and metal weight.

All electrodes were tested for two cycles at increasingly cathodic potentials, between −0.5 and −1.1 V vs RHE. Figure 3A shows a typical chronoamperometry measurement of the Cu_0.14_Zn_0.86_O electrode. The current is stable at each potential within the 30 min testing time. Only at the start of the measurement an increased cathodic current is observed. This is likely related to the reduction of copper and zinc oxide species, which is thermodynamically favorable in this potential range. Since this feature is much less pronounced in the ZnO sample (Figure S4), the main contribution here arises from copper oxide reduction. This implies that copper oxide reduction takes place on much shorter time scales than zinc oxide reduction.

Figure 3B shows a comparison of the current densities of the different catalysts at a similar applied potential of −0.9 V vs RHE. All current densities are normalized by the mass of copper and zinc metal present on the electrodes to account for the slightly different loadings summarized in Table 1. A clear trend can be observed, with a higher relative Cu to Zn content resulting in a higher cathodic current density. This shows that copper is a more active reduction catalyst than zinc. The geometric current densities without normalization by metal weight are given in Figures S6–S8. Note that the main difference upon normalization is for the CuO electrode, which shifts the most since it has the lowest metal weight loading.

Although the total current density is a good descriptor of the catalyst activity, it provides no information on the type of products formed. For all electrodes, the main reaction products are H_2_ and CO. Figure 3C,3D show their respective partial current densities as a function of potential. Other reaction products include CH_4_, C_2_H_4_, and a number of liquid products like formate, ethanol, acetate, and 1-propanol, all of which are formed mainly on the CuO and Cu_0.75_Zn_0.25_O electrodes as shown in Figure S5 and summarized in Table S2.

From Figure 3C, it follows that the activity for H_2_ production depends strongly on the Cu:Zn ratio, with a higher relative copper content generally resulting in more H_2_ production. The only exception is the pure CuO electrode, which shows a lower H_2_ partial current density at more cathodic potentials than the Cu_0.75_Zn_0.25_O electrode. These results explain to a large extent why the total cathodic current in Figure 3B increases with a higher relative Cu content, whereas the total current is similar for the CuO and Cu_0.75_Zn_0.25_O electrodes.

On the other hand, the activity for CO production as shown in Figure 3D follows a different trend. Upon addition of copper to ZnO, the CO activity increases, with the Cu_0.43_Zn_0.57_O electrode showing an optimum production of CO. This effect does not persist for higher Cu:Zn ratios, which show lower CO partial current densities with increasing relative copper content. Overall, all bimetallic electrodes show a higher CO activity than the monometallic CuO and ZnO electrodes when normalized by catalyst metal weight loading. The optimum CO activity for the Cu_0.43_Zn_0.57_O electrode regardless of normalization is remarkable, and indicates the presence of a synergistic interaction between copper and zinc.

**Figure 4:**
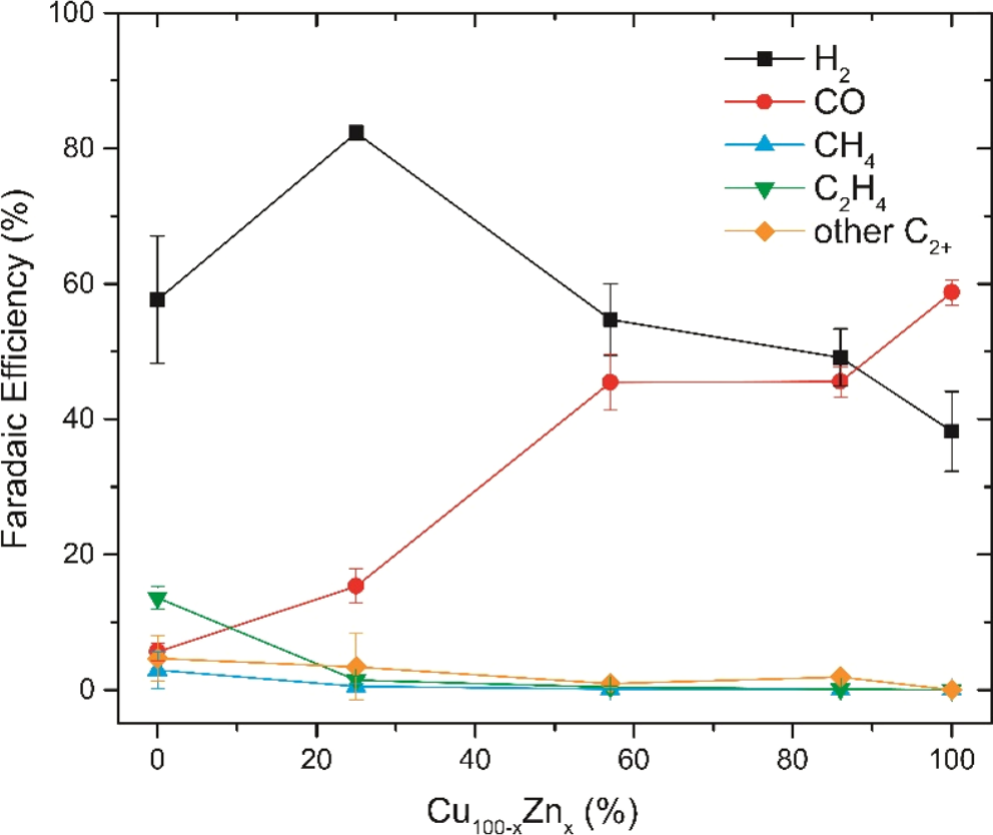
Faradaic efficiency as a function of Cu:Zn ratio at −0.95 V. Other C_2+_ products include ethanol, acetate, and 1-propanol.

The selectivity of the different catalysts at −0.95 V as a function of Cu:Zn ratio are summarized in Figure 4. As previously discussed, H_2_ and CO are the main products observed for all of the catalysts. The only exception is the pure CuO catalyst, which has a low CO FE but instead produces hydrocarbon products such as CH_4_ and C_2_H_4_ to a significant extent. It is also clear from this graph that the higher the relative Zn content, the higher the selectivity to CO.

Interestingly, at this potential, the FE for CH_4_, C_2_H_4_, and other C_2+_ products does not amount to more than 5% for the bimetallic catalysts, whereas it is significantly higher for the pure CuO electrode. Most notable is that the presence of ZnO suppresses the formation of C_2_H_4_. This can also be observed in Table S2, which shows that the onset potential for C_2_H_4_ production is more cathodic for the bimetallic Cu_0.75_Zn_0.25_O catalyst (−0.85 V) than that for CuO (−0.73 V). Additionally, Figure S5B shows that the formation of C_2_H_4_ is larger on CuO than that on the bimetallic catalysts at similar potentials. Surprisingly, the selectivity to C_2+_ oxygenates like acetate, ethanol and 1-propanol is also higher on CuO than on the bimetallic catalysts, in contrast to other studies.^19,20^

This is remarkable, as this finding contradicts what is expected based on CO-spillover effects. It also indicates that although the electrodes consisted of spatially separated CuO and ZnO before catalysis, both metals are able to interact during the CO_2_RR and are hence in intimate contact, implying mobility of Zn and/or Cu species during catalysis. These effects will be discussed in further detail in the following sections.

### Catalyst Stability

**Figure 5:**
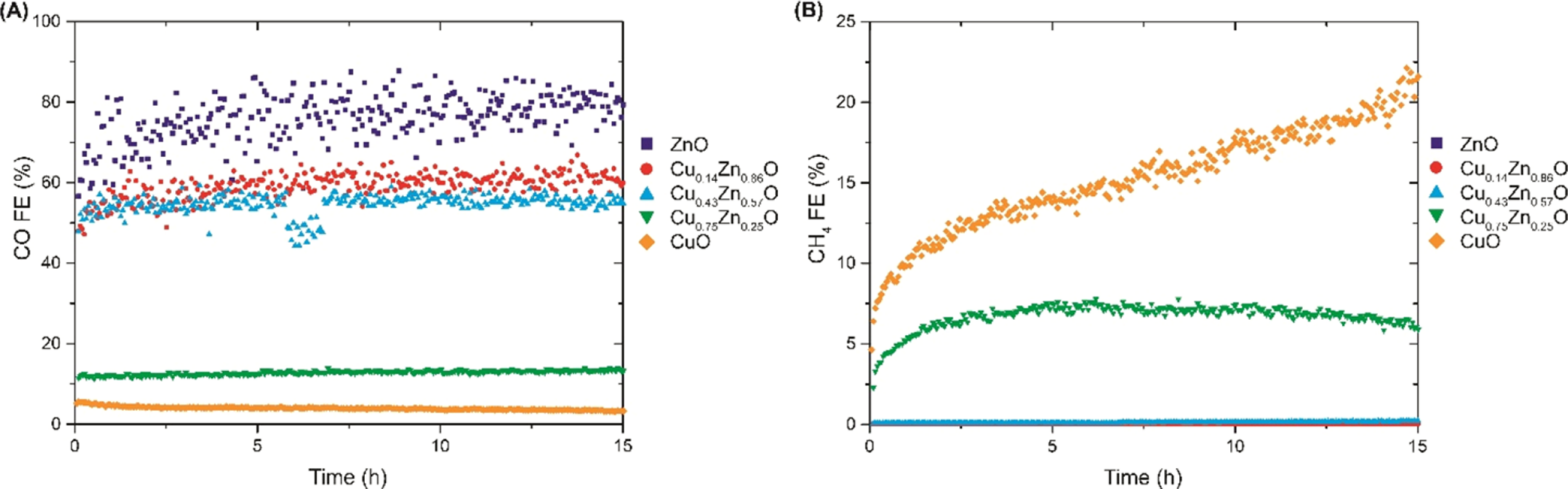
Stability tests of all electrodes at −1.0 V, showing the evolution of the CO (A) and CH_4_ (B) FE over time.

It is well-known that both Cu- and Zn-based electrocatalysts are not structurally stable during the CO_2_RR. Therefore, it is important to also investigate the changes in both the structure and catalytic performance as a function of time. To this end, stability studies were performed at a fixed potential of −1.0 V. The CO FE of all catalysts are shown in Figure 5 as a function of time for 15 h. All catalysts show stable CO selectivity over the testing duration. A slight drop is observed in both the CO and H_2_ FE of the Cu_0.43_Zn_0.57_O electrode in between 6 and 7 h. This might be explained by a temporary issue with the gas flow, as no change in the potentiostat data is observed at this time, and the CO:H_2_ ratio remains constant. Therefore, this is not an effect of the electrode.

Most notably, ZnO actually shows an increase in the CO FE in the first few hours. This effect is also present for the Zn-rich bimetallic catalysts, although less pronounced. Given that the stability studies were carried out after testing at the different cathodic potentials, we think that the most significant changes on these catalysts happen relatively quickly at the start of all measurements. These changes are likely related to the reduction of copper and zinc oxides, accompanied by catalyst restructuring. Zinc oxide reduction is slower than copper oxide reduction, explaining the longer time required for achieving a stable CO FE on ZnO. After reduction, a more stable catalyst active state is formed, resulting in the observed stable CO selectivity.

Looking at the evolution of the CH_4_ FE over time, it is clear that the CuO-rich catalysts exhibit a less stable catalytic performance in terms of their product selectivity toward CH_4_ than CO. Especially for the CuO electrode, a ca. 3-fold increase of the CH_4_ FE is observed during 15 h of testing. On the other hand, for the Cu_0.75_Zn_0.25_ electrode, the CH_4_ FE is relatively more stable over time, whereas the other catalysts show no significant CH_4_ production. For CuO, the increase in CH_4_ FE is accompanied by both a decrease in H_2_ FE and increase in C_2_H_4_ FE in the first hours of testing as shown in Figure S9, and thus an improvement in CO_2_RR efficiency. However, at longer testing times, the increasing FE of CH_4_ goes at the cost of C_2_H_4_. These changes indicate that the CuO catalyst is not stable, and that it is important to take long-term stability tests into account when evaluating electrocatalyst performance.

The structural stability of the catalysts was investigated by ICP and SEM-EDX measurements on the electrodes after catalysis. The ICP results in Table S3 show no significant change in Cu:Zn ratio after catalytic testing. However, some loss of metal is observed. This is likely due to particle detachment from the binder-free electrodes. We believe metal dissolution is minimal, as the different solubilities of copper and zinc would have affected the Cu:Zn ratio in this case.

**Figure 6:**
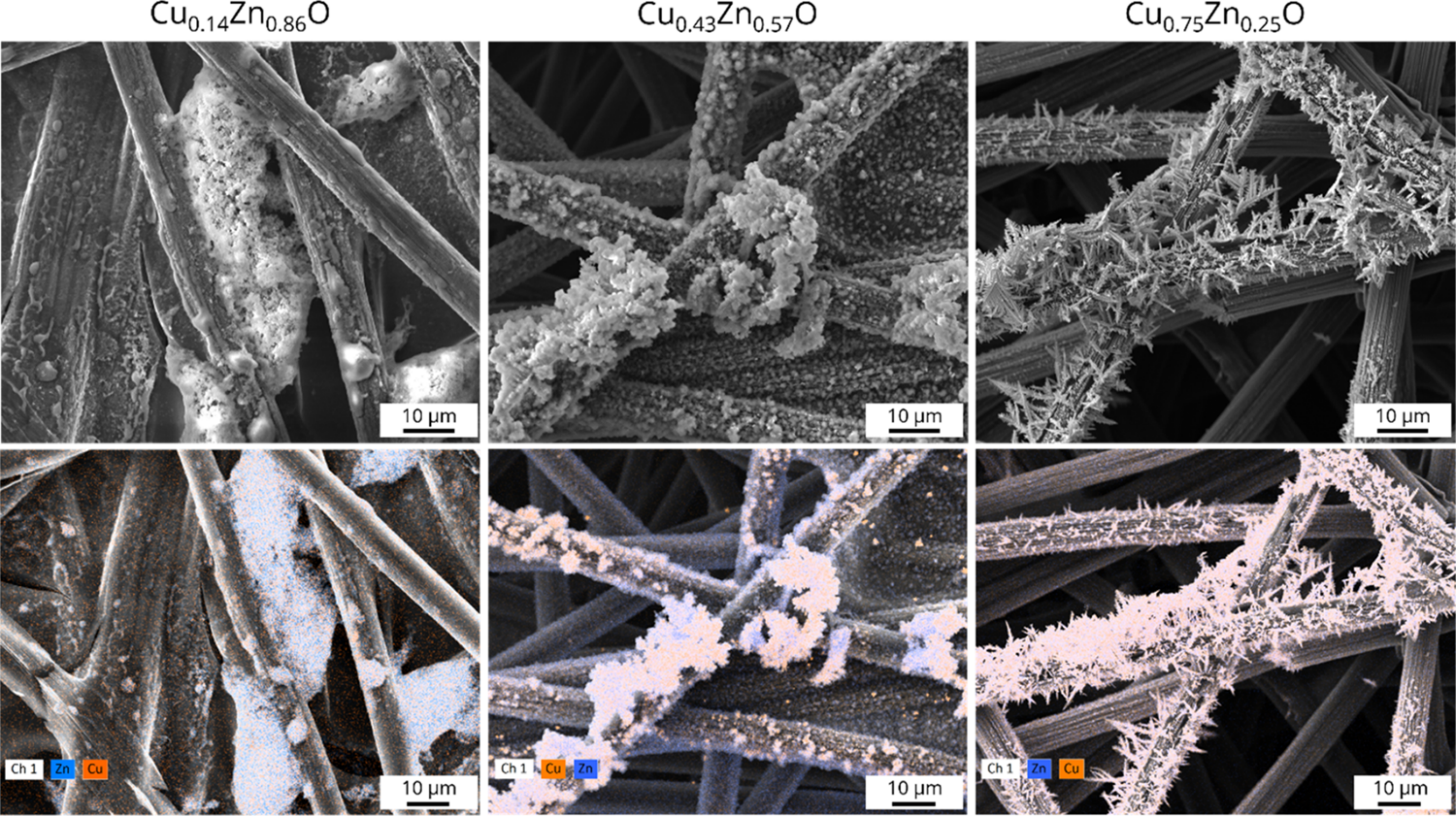
SEM images (top) and corresponding EDX maps (bottom) of the three bimetallic CuO–ZnO catalysts after testing, with Cu indicated in orange and Zn in blue.

Figures 6, S11, and S12 show the SEM-EDX images of the CuO–ZnO catalysts after testing. The results show that both the CuO and ZnO particles have lost their initial shape. Instead, smaller fragmented particles as well as larger amorphous agglomerates are observed for all electrodes. Additionally, dendritic structures are observed on the copper-rich Cu_0.75_Zn_0.25_O sample. These findings are in line with other studies on electro-reconstruction of copper^26,27^ and zinc^10,16^-based structures, which show that fragmentation and subsequent agglomeration readily take place under CO_2_RR conditions. This likely happens through a dissolution-redeposition mechanism.^26^ Recently, it has been argued that the soluble transient copper species that drive electro-reconstruction are copper carbonyls and oxalates with copper in a +1 oxidation state.^28^

Before catalytic testing, the electrodes consisted of spatially separated CuO and ZnO structures. However, because of significant reconstruction, such a spatial separation is no longer visible in the SEM-EDX maps after catalysis. It should be noted that the spatial resolution of SEM-EDX measurements is limited to around 1–2 μm, making it impossible to assess the proximity of copper and zinc on smaller length scales using this technique.

For this reason, TEM-EDX maps were acquired, as shown in Figure S13. Interestingly, nanoscale mixing is observed in these images, especially in the cases of the Cu_0.14_Zn_0.86_O and Cu_0.43_Zn_0.57_O catalysts. These images show the formation of intimately mixed Cu–Zn phases, which suggests that alloying occurs under CO_2_RR reaction conditions, even though the initial electrodes consisted of spatially separated particles. However, this mixing is not entirely homogeneous, as for both the Cu_0.14_Zn_0.86_O and Cu_0.75_Zn_0.25_O catalysts, more Zn-rich and more Cu-rich regions are observed. We think that the extent of mixing is related to the oxidation state of both metals under the CO_2_RR conditions, with intimate mixing requiring full reduction of copper and zinc oxides. Conversely, the presence of segregated domains indicates incomplete reduction of the initial copper and/or zinc oxides.

The fact that the bimetallic electrodes showed higher CO partial current densities than the monometallic ones and that the addition of zinc to copper increased the cathodic potentials required for production of hydrocarbons strongly suggests that copper and zinc interact. This is remarkable because it is generally assumed that intimate mixing (at the atomic-scale) of the starting materials is required for synergistic effects in bimetallic electrocatalysts. In contrast, the electrodes used in this work consisted of spatially separated CuO and ZnO particles initially. Importantly, the EDX maps show that intimate mixing is not a prerequisite for the starting materials because it takes place under reaction conditions. As such, these results underline the importance of catalyst characterization after testing because significant restructuring can occur during CO_2_RR, which in turn could strongly affect the electrocatalytic CO_2_RR performance. To better understand the complex interplay between catalyst oxidation state, structure, and catalytic performance, *in situ* XAS measurements were performed.

### *In Situ* XAS Studies on the Bimetallic CuO–ZnO Catalysts

**Figure 7:**
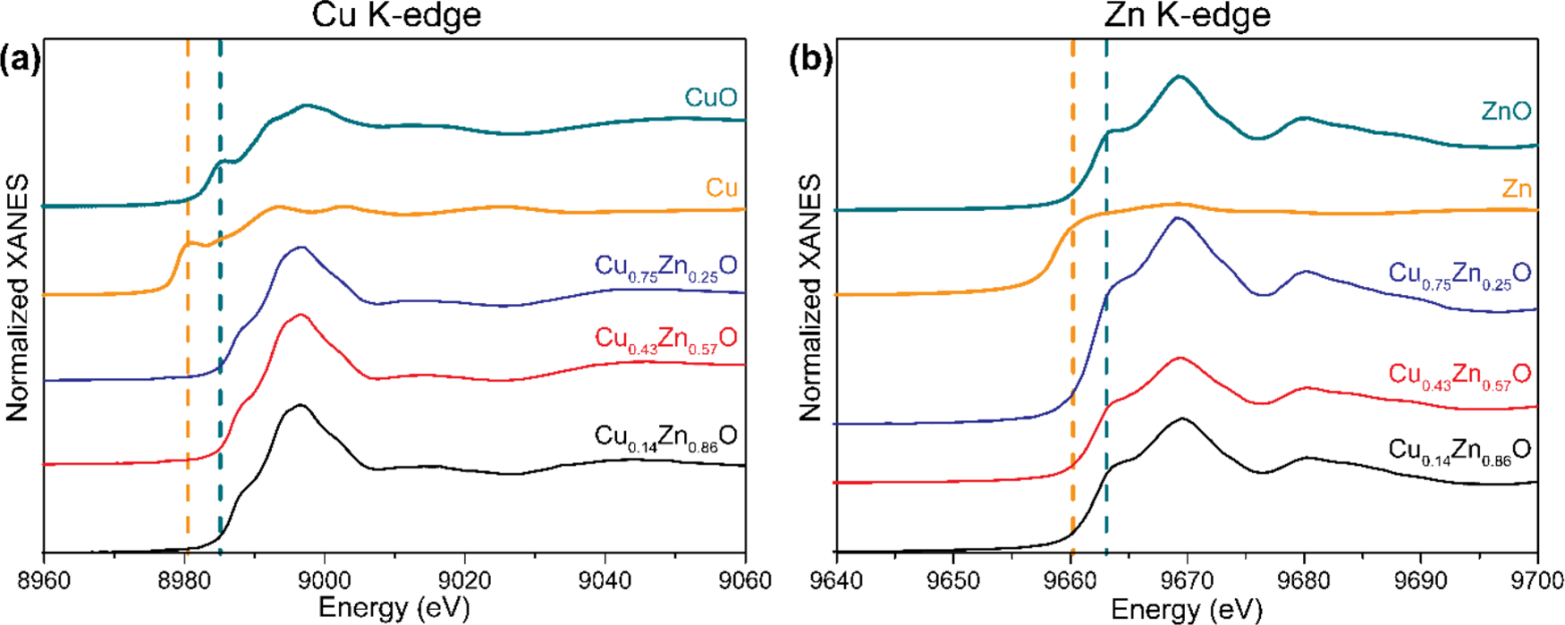
Cu K-edge (a) and Zn K-edge (b) normalized XANES spectra of fresh CuO–ZnO catalysts and the corresponding normalized XANES spectra of reference Cu, CuO, Zn, and ZnO materials. Shoulder peak positions of Cu and Zn reference spectra are indicated by yellow dotted lines, of CuO and ZnO by green ones.

To gain further insight into the chemical state and structure of the catalysts under operating conditions, *in situ* X-ray absorption spectroscopy (XAS) measurements were performed at the LISA beamline at the ESRF in Grenoble. First, the *ex-situ* spectra of the fresh catalysts will be discussed. Figure 7a,7b shows the normalized Cu and Zn K-edge X-ray absorption near edge structures (XANES) spectra for the fresh bimetallic CuO–ZnO catalysts with different Cu:Zn ratios together with those of the Cu, CuO, Zn, and ZnO references. The Zn K-edge absorption peaks of the fresh catalysts match those of the ZnO reference. This is in agreement with the XRD data, which shows the presence of ZnO phases. Additionally, detailed analysis of the Zn K-edge by comparison to reference spectra (Figure S14) confirms that Zn is in a + 2 oxidic state and hexagonal in structure. The normalized Cu K-edge XANES spectra for the CuO–ZnO catalysts do not resemble closely that of CuO. This is possibly due to a more amorphous nature of the copper species, the presence of copper hydroxides, or an interaction with nearby ZnO.

**Figure 8:**
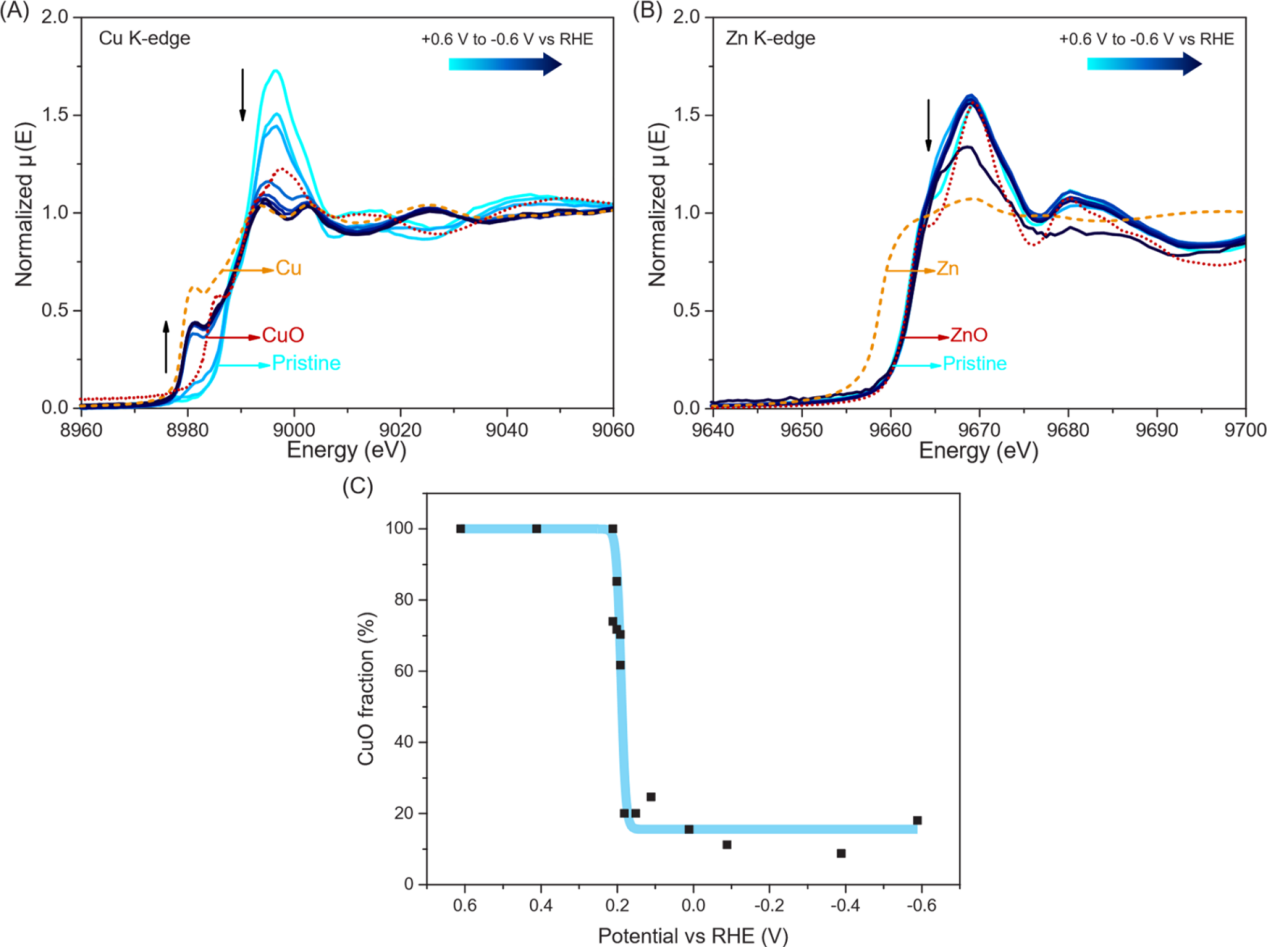
Normalized *in situ* (A) Cu K-edge and (B) Zn K-edge XANES spectra of Cu_0.14_Zn_0.86_O and Cu, CuO, Zn, and ZnO references in CO_2_-saturated 0.1 M KHCO_3_ under CO_2_RR conditions at potentials of +0.6 to −0.6 V vs RHE. (C) Results of linear combination analysis on Cu K-edge XANES results of Cu_0.14_Zn_0.86_O catalyst for determination of the CuO fraction at different applied potentials.

Both the Cu and Zn K-edge XANES spectra in Figures 8, S16, and S18 show little differences between the three different catalysts, even though they differ in terms of elemental composition. Hence, the following discussions focus only on the Cu_0.14_Zn_0.86_O catalyst.

*In situ* XAS measurements were carried out at different potentials to obtain information on the changes in the chemical state and structure of the Cu_0.14_Zn_0.86_O catalyst under CO_2_RR conditions. Figure 8A shows the evolution of the Cu K-edge spectra in the potential range of 0.6 to −0.6 V. It should be noted here that these potentials are more anodic than the observed onset potential for the CO_2_RR (around −0.7 V) because of experimental difficulties with, for example, bubble formation at more cathodic potentials. Upon applying increasingly negative potentials, the Cu K-edge absorption shifts toward a lower energy, and the white line intensity decreases. Whereas the spectra initially resemble the CuO reference, the spectra at the most negative potentials recorded here resemble that of the Cu reference most closely. No large changes in the XANES spectra are observed for the potentials below 0.1 V, suggesting that copper reduction is complete. Interestingly, however, Cu reduction does not seem complete even at −0.6 V, since no complete agreement with the Cu reference spectrum is obtained.

To quantify the extent of copper oxide reduction, a linear combination analysis is applied to compare the obtained Cu K-edge XANES spectra at different potentials to the Cu and CuO reference spectra. The results of this analysis are shown in Figure 8C. Figure 8C shows that copper oxide reduction takes place mainly between 0.2 and 0 V. Interestingly, this linear combination analysis indicates that not all copper on our electrode is fully reduced, and some oxidized species persist even at −0.6 V. A possible explanation for this behavior is close contact and interaction with ZnO. Such an electronic interaction has also been suggested by others for electrodeposited CuZn electrodes, showing that this interaction is relevant regardless of the difference in initial oxidation state.^29^

Figure 8B shows the Zn K-edge EXAFS spectra obtained at potentials ranging from 0.6 to −0.6 V vs RHE. In contrast to the Cu K-edge, the changes in these spectra are much less pronounced, and all spectra strongly resemble that of the ZnO reference. This is logical given the thermodynamic reduction potential of zinc oxide being more cathodic, namely −0.83 V at pH 6.8. Interestingly, however, the −0.6 V spectrum features a decrease in the white line intensity, although no shift in absorption energy is observed. This indicates partial reduction of ZnO, which is likely enabled by its electronic interaction with nearby reduced Cu species. Not only does this affect the electronic structure of the ZnO, but also a structural effect is observed, with zinc partially changing from a tetrahedral to octahedral configuration as shown in Figure S15.

**Figure 9:**
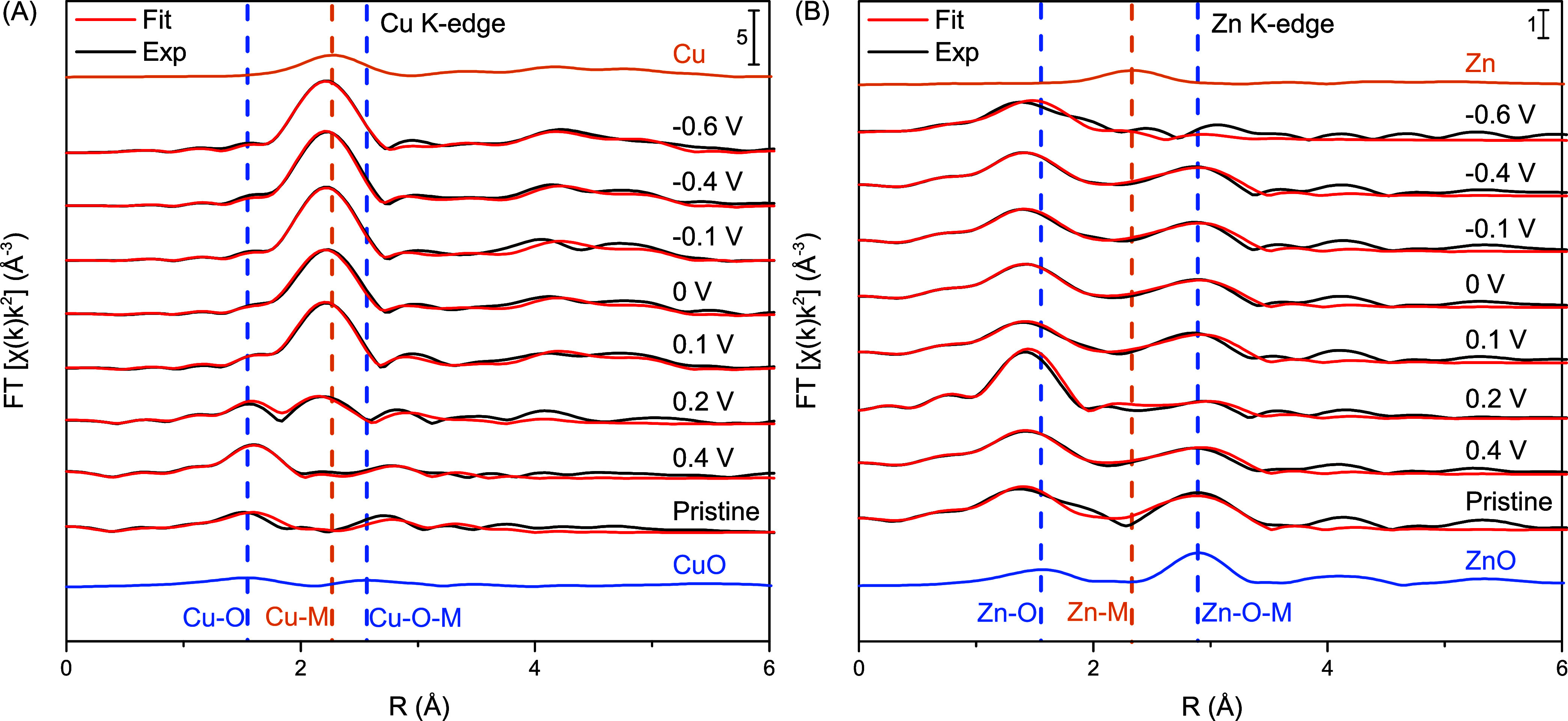
Fourier-transformed (FT) EXAFS data at different potentials for the (A) Cu K-edge and (B) Zn K-edge of the Cu_0.14_Zn_0.86_O catalyst under CO_2_RR conditions. Note that these data are not phase corrected, meaning that the FT-EXAFS peak positions do not correspond to the true RDF peak positions and bond lengths. Black lines show the experimental data; red lines show the corresponding fits.

Figure 9 shows the Fourier-transformed (FT) Cu K-edge and Zn K-edge EXAFS spectra of the Cu_0.14_Zn_0.86_O catalyst over a range of applied potentials under CO_2_RR conditions. The fitting results corresponding to each spectrum are also shown, with the quantitative data summarized in Tables S3 and S4. The FT-EXAFS spectra of the fresh catalysts show contributions of both Cu–O and Zn–O bonds at 1.5 Å for both the Cu K-edge and Zn K-edge (not corrected for phase shift). The phase corrected Cu–O and Zn–O bond lengths are given in Tables S3 and S4, being 1.99 and 1.93 Å, respectively. They correspond to the expected values for bulk CuO and ZnO of 1.94 and 1.97 Å.^30^ Furthermore, contributions of second-shell Cu–O–M and Zn–O–M bonds can be observed in the spectra of the fresh catalysts at 2.6 and 2.9 Å, respectively. The corresponding phase corrected bonds lengths are given in Tables S3 and S4, being 3.02 and 3.23 Å, respectively. Although the second-shell Zn–M bond is in line with the expected value of 3.25 Å for bulk ZnO, the second-shell Cu–M bond is slightly higher than the expected 2.84 Å.^30^ This value lies between the values of the CuO and ZnO reference materials, indicating intimacy between the copper and zinc atoms.

Upon application of increasing cathodic potentials, clear changes in the Cu K-edge FT-EXAFS spectra can be observed. The contributions of both the first-shell Cu–O and second-shell Cu–O–M bonds decrease in intensity and can no longer be observed at 0 V and more cathodic potentials. At the same time, a new peak appeared at 2.2 Å. This can be assigned to a first-shell Cu–M bond, similar to that observed for the Cu foil. It should be noted that it is impossible to discriminate between Cu–Cu and Cu–Zn because they are neighbors in the periodic table and, hence, have similar scattering functions.

Table S3 provides the quantitative analysis results of the Cu K-edge EXAFS data. At 0.1 V and more negative potentials, the second-shell Cu–O–M contribution disappears, while the coordination number corresponding to the Cu–O bond decreases to below 1. This is in line with the previously discussed XANES data, which showed a strong but incomplete reduction of copper oxide, even at −0.6 V.

Changes in the FT-EXAFS spectra of the Zn K-edge are less pronounced than those of the Cu K-edge. A clear decrease in intensity for both the Zn–O and Zn–O–M contributions is observed only at −0.6 V, as well as the appearance of a new peak where the first-shell Zn–M bond is expected, based on the Zn foil reference. This indicates that ZnO reduction partially takes place at −0.6 V. This remarkable finding is in line with the previously discussed XANES data, again indicating that the presence of nearby reduced Cu species enables the (partial) reduction of ZnO.

Based on the findings of the *in situ* XANES and EXAFS analysis, combined with the *ex-situ* SEM and TEM analyses, we deduce that copper and zinc clearly show electronic interactions despite being spatially separated before catalysis. This indicates the high mobility of both copper and zinc species under CO_2_RR conditions. Furthermore, the presence of copper enables zinc reduction above its thermodynamic reduction potential, whereas the presence of zinc oxide prevents the full reduction of copper. As such, combining metals with different reduction potentials is a promising strategy for tuning the electrocatalyst oxidation state under the CO_2_RR conditions.

The electronic interactions between copper and zinc play an important role in their electrocatalytic performance. Specifically, all bimetallic CuO–ZnO electrodes showed improved activity for CO as compared to the pure CuO and ZnO ones. Based on our findings, we suspect that the addition of copper increased the amount of catalytically active Zn species that benefit the CO activity. Even though the *in situ* XAS measurements were performed at a potential range where CO_2_RR activity is less pronounced, a detailed look at Figure 3 indicates a significant decrease in HER activity upon addition of ZnO to CuO at −0.6 V, more than would be expected based on their respective amounts. Additionally, we found that the bimetallic catalysts showed surprisingly little ethylene formation. Based on the *in situ* XAS and *ex-situ* microscopy results, we rationalize these findings by the interaction of copper with nearby zinc oxide, which prevents the full reduction of copper. Indeed, it has been reported for copper-only catalysts using pulse experiments that the presence of cationic copper species lowers the selectivity toward C_2_H_4_.^31^ However, the effect of the oxidation state has been a topic of strong debate, with some studies also arguing that the copper oxidation state does not play an important role in electrodeposited CuZn-based systems.^29^ We think that by starting from CuO and ZnO rather than electrodeposited CuZn in which the majority of species is already in a reduced form before catalytic testing, the effect of the copper and zinc oxidation state is more pronounced in our catalytic data.

## Conclusions

We studied the electrocatalytic reduction of CO_2_ on oxide-derived CuO–ZnO electrodes with tunable compositions. The use of hydrothermally synthesized CuO–ZnO electrodes enabled us to study the effects of the initial catalyst structure, composition, and oxidation state on electrocatalytic CO_2_RR performance. Whereas the as-prepared electrodes consisted of spatially separated ZnO and CuO particles on carbon paper, strong restructuring and atomic mixing took place under CO_2_RR conditions. This leads to an intimate contact between copper and zinc (oxide) and thereby a change in the catalyst activity and selectivity. Specifically, the higher the copper content, the higher the overall activity. Interestingly, all of the bimetallic electrodes showed higher CO production when corrected for the weight of metal than the monometallic electrodes, with an optimum for the Cu_0.43_Zn_0.57_O electrode. Furthermore, addition of zinc to copper decreased the C_2_H_4_ selectivity, in contrast to what would be expected based on CO-spillover effects.

Especially remarkable is the interplay between CuO and ZnO proximity and oxidation state during the CO_2_RR and the consequent effect on the electrocatalytic performance. Using *in situ* XAS measurements, we observe that CuO is readily reduced at potentials more negative than 0.1 V. Surprisingly, however, the reduction is not complete even at −0.6 V because of the interaction with nearby ZnO. On the other hand, ZnO is partially reduced at −0.6 V which is below its thermodynamic reduction potential. This shows that copper facilitates the reduction of ZnO, leading to more catalytically active species for CO, while stabilization of the cationic copper species by nearby partially reduced ZnO suppresses the ability of Cu to make ethylene. These results clearly demonstrate that the initial structure of electrocatalysts is of minor importance to the catalytic performance due to profound structural modifications under the reaction conditions. Our work also reveals that electronic modification via interaction with metal oxide species with different reducibility in oxide-derived bimetallic catalysts is a promising approach to design electrocatalysts with improved activity and selectivity for electrochemical CO_2_ reduction.

## Supporting Information Available

Electrochemical cell schematics, XRD patterns, electrochemical data, SEM and TEM images and TEM-EDX maps after catalytic testing, *in situ* XANES and EXAFS data (PDF)
