# Supplementary material for: Synthesis and Catalytic Performance of Bimetallic Oxide-Derived CuO–ZnO Electrocatalysts for CO2 Reduction
Source: ACS Catal. 2024 Jul 2;14(14):10701–11. doi: 10.1021/acscatal.4c01575 (PMC11264205; doi:10.1021/acscatal.4c01575)
Supplement: Supplementary file 1 — cs4c01575_si_001.pdf [file cs4c01575_si_001.pdf]

Supporting Information

Synthesis and catalytic performance of bimetallic oxide-derived CuO-ZnO electrocatalysts for CO_2 reduction

Matt L.J. Peerlings[1], Kai Han[1], Alessandro Longo[2,3], Kristiaan H. Helfferich[1], Mahnaz Ghiasi[1], Petra E. de Jongh[1]*, Peter Ngene[1*]

1. Materials Chemistry and Catalysis, Debye Institute for Nanomaterials Science, Utrecht University, 3854 CG, Utrecht, The Netherlands

2. European Synchrotron Radiation Facility (ESRF), 71, Avenue des Martyrs, Grenoble F-38000, France

3. Istituto per lo Studio dei Materiali Nanostrutturati (ISMN)-CNR, UOS Palermo, via Ugo La Malfa 153, Palermo 90146, Italy

[*]E-mail: P.E.dejongh@uu.nl and P.Ngene@uu.nl

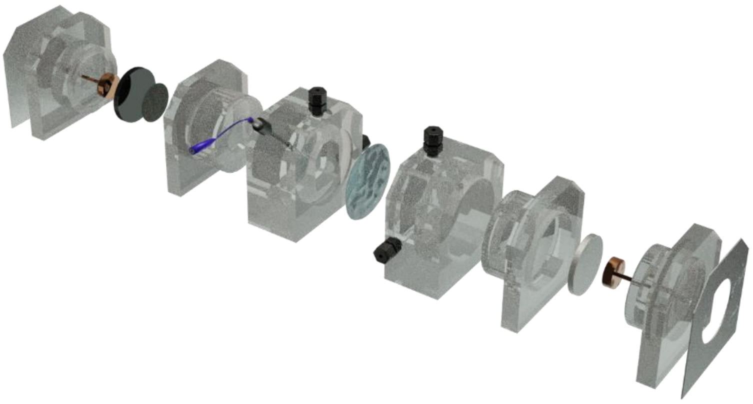

Figure S1. Custom-made H-type electrochemical cell used for catalytic tests. From left to right: glassy carbon current collector and carbon paper containing the CuO-ZnO catalyst, cathode compartment with Ag/AgCl reference electrode, Fumasep FAA-3-PK-130 anion exchange membrane, anode compartment, and carbon paper and glassy carbon counter electrode.

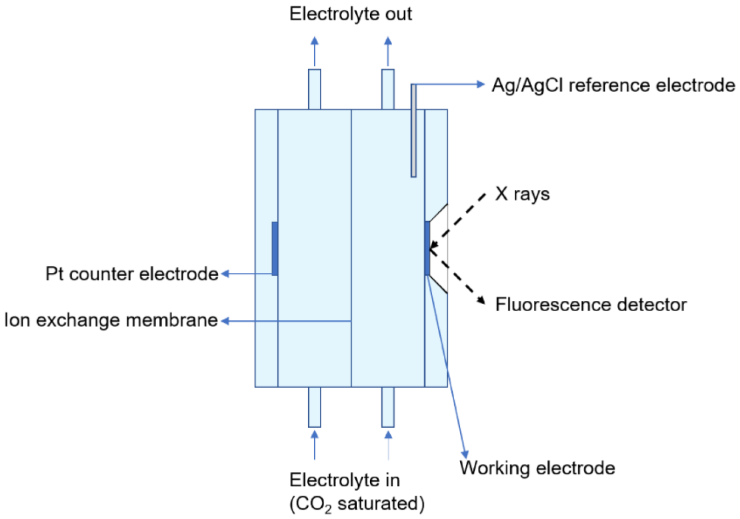

Figure S2. Schematic illustration of flow cell designed for *in-situ* XAS and high current density measurements.

Table S1. pH of $Cu_{1-x}Zn_xO$ synthesis solutions before and after heating step.

**Sample**	pH before	pH after
**ZnO**	5.84	5.68
**$Cu_{0.14}Zn_{0.86}O$**	5.06	5.80
**$Cu_{0.43}Zn_{0.57}O$**	5.14	6.08
**$Cu_{0.75}Zn_{0.25}O$**	4.81	5.76
**CuO**	4.89	5.61

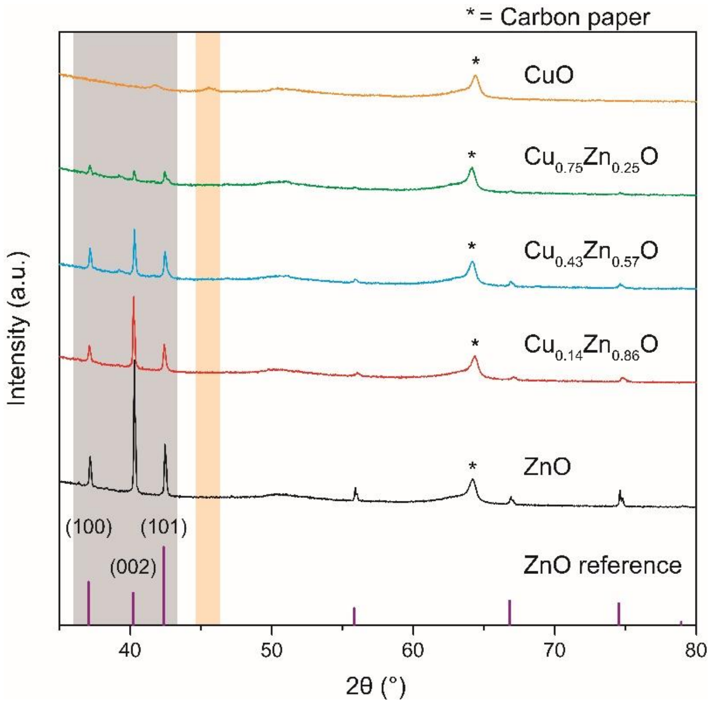

Figure S3. XRD patterns of the $Cu_{1-x}Zn_xO$ catalysts and ZnO reference pattern. Shaded gray and yellow areas indicate the regions that show the respective ZnO and CuO reflections most clearly. The higher relative intensity of the (002) peak indicates a preferential growth direction of ZnO, likely caused by a structure directing effect of the hexamethylenetetramine (HMT) molecule present in the hydrothermal synthesis solution.

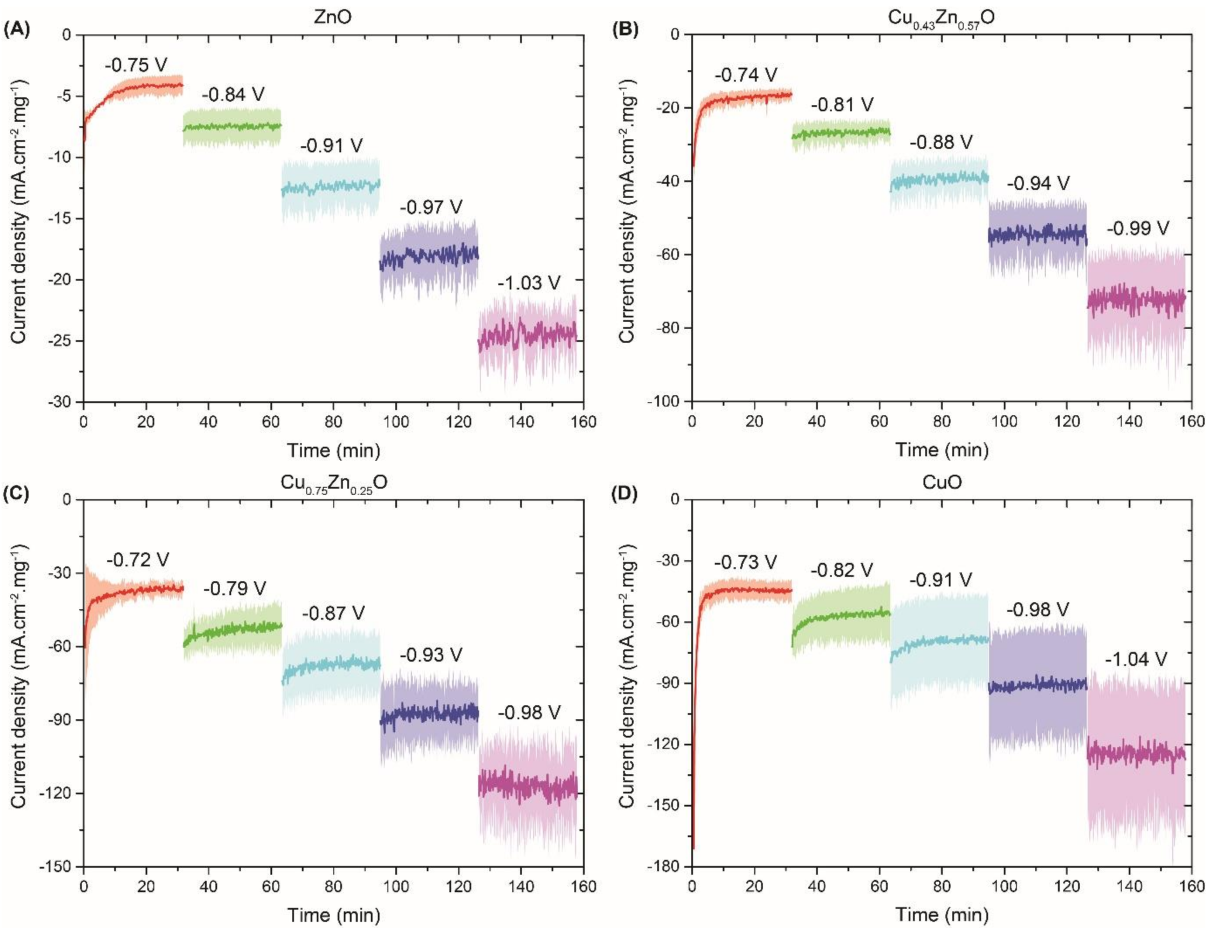

Figure S4. Chronoamperometry data of (A) ZnO, (B) $Cu_{0.43}Zn_{0.57}O$, (C) $Cu_{0.75}Zn_{0.25}O$ and (D) CuO catalysts at five consecutive applied potentials. Currents are normalized by geometric surface area and metal weight. The indicates lines are an average of three independent measurements, with the shaded areas indicating the standard deviation.

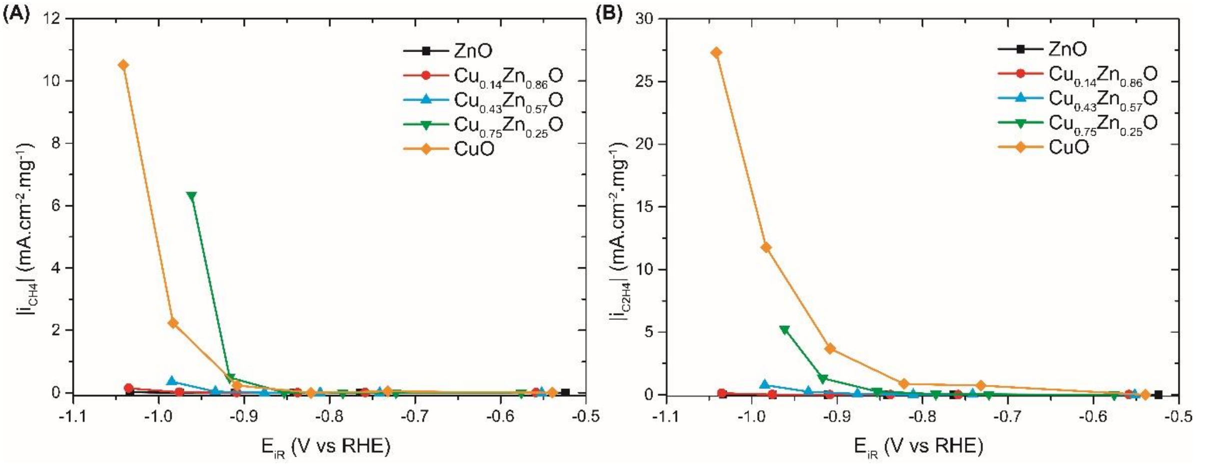

Figure S5. Partial current density for (A) CH_4 (B) C_2H_4 production of the five different catalysts tested, normalized by geometric surface area and metal weight. The indicated lines are an average of three independent measurements.

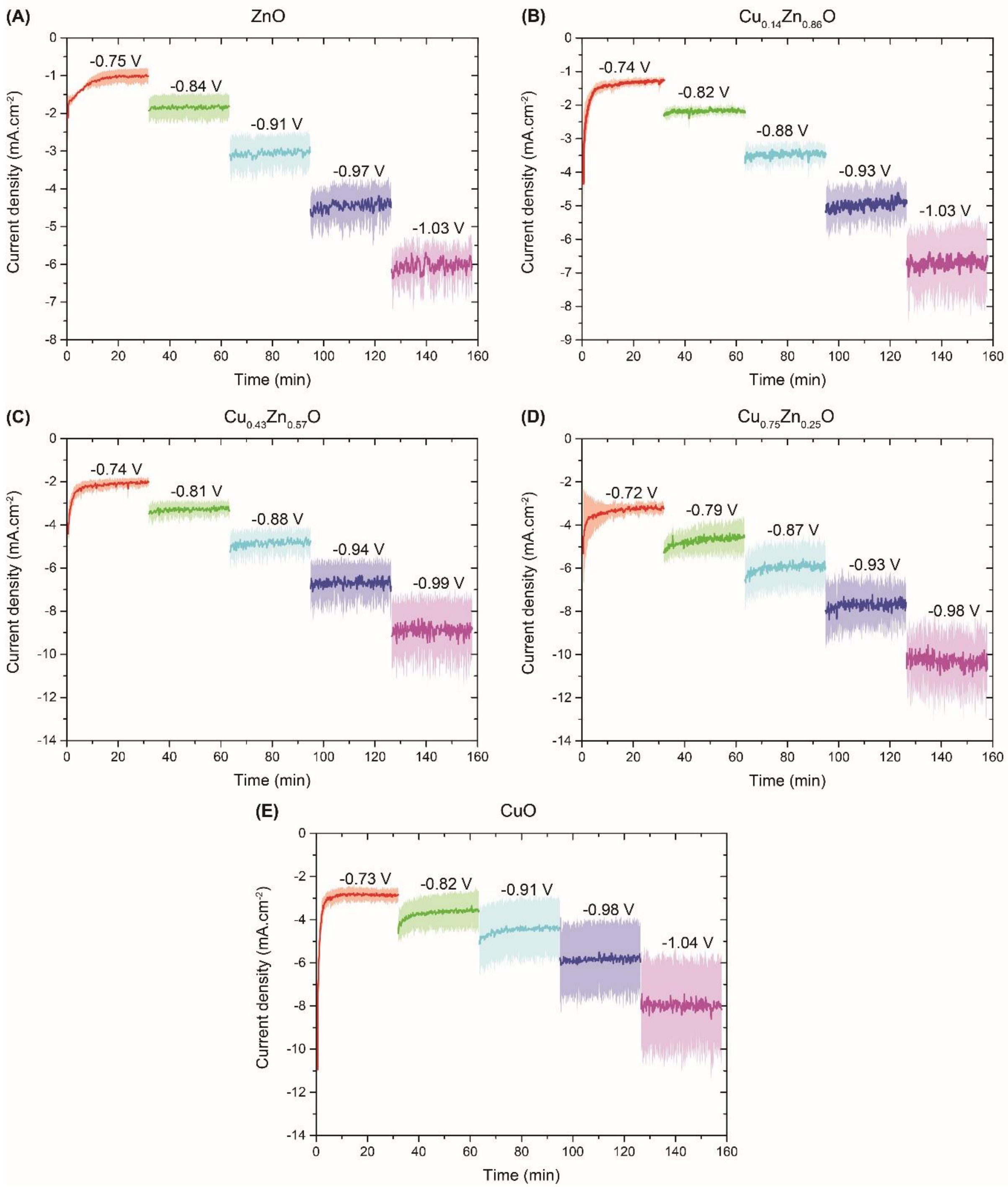

Figure S6. Chronoamperometry data of (A) ZnO, (B) $Cu_{0.14}Zn_{0.86}O$, (C) $Cu_{0.43}Zn_{0.57}O$, (D) $Cu_{0.75}Zn_{0.25}O$ and (E) CuO catalysts at five consecutive applied potentials. Currents are normalized by geometric surface area only. The indicates lines are an average of three independent measurements, with the shaded areas indicating the standard deviation.

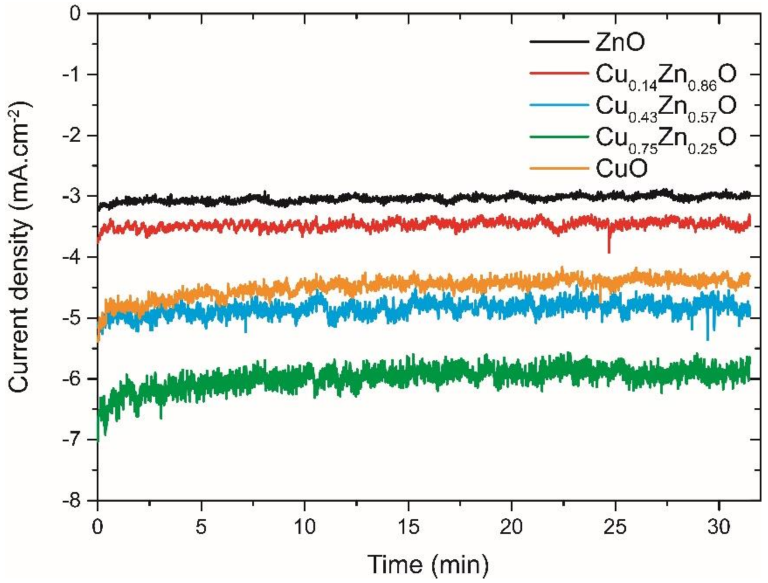

Figure S7. Geometric current density of all electrodes at -0.9 V vs RHE, normalized by geometric surface area only. The indicated lines are an average of three independent measurements.

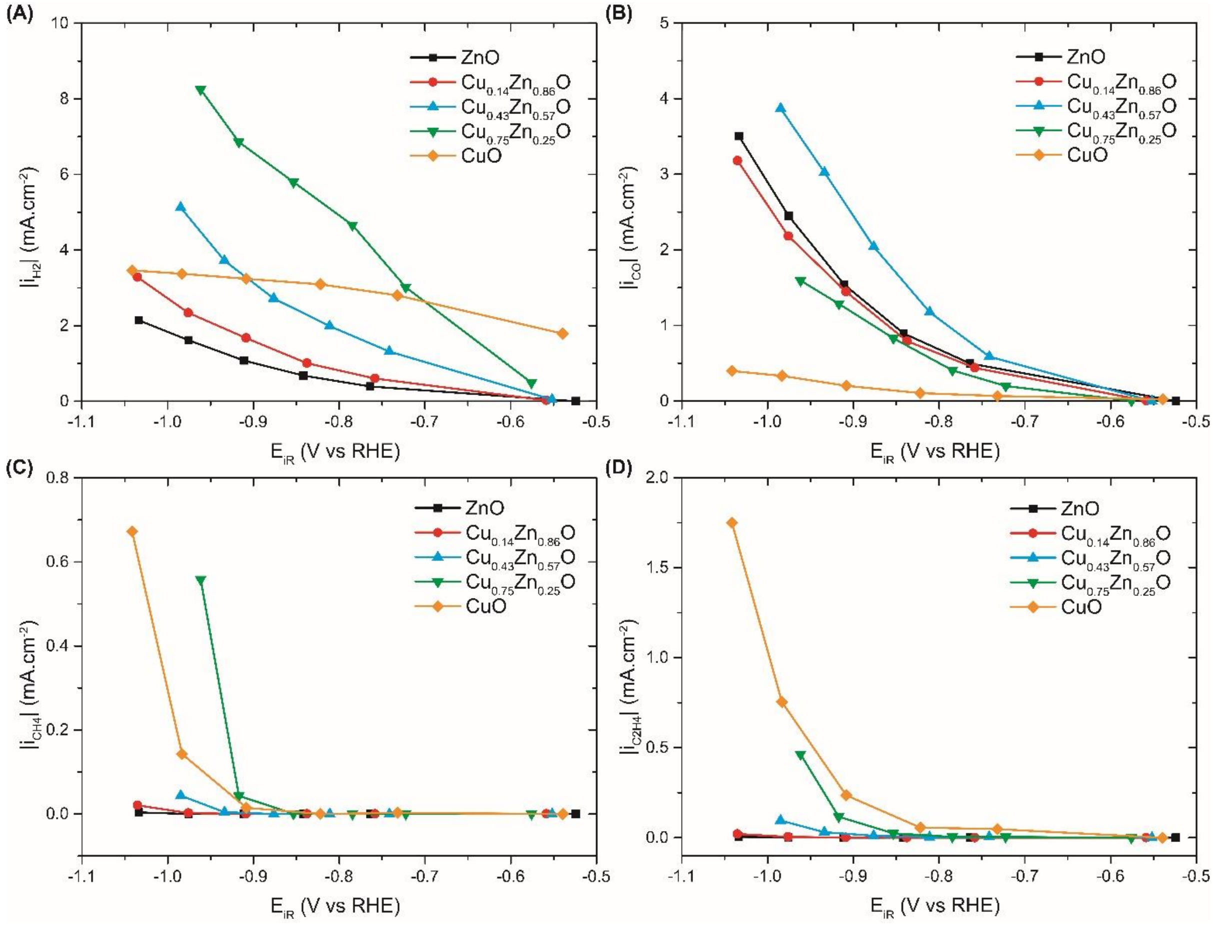

Figure S8. Partial current density for (A) H_2, (B) CO, (C) CH_4 and (D) C_2H_4 production of the five different catalysts tested, normalized by geometric surface area only. The indicated lines are an average of three independent measurements.

Table S2. Faradaic Efficiency of CuO-ZnO based electrodes.

		Faradaic Efficiency (%)							
**Sample**	E_{iR} vs RHE (V)	**H_2**	**CO**	**CH_4**	**C_2H_4**	**HCOOH**	**C_2H_5OH**	**Other[1]**	**Total**
**ZnO**	-0.76	39.8 ± 8.5	53.5 ± 4.9	0.0	0.0	0.0	0.0	0.0	93.3
	-0.84	37.9 ± 5.7	51.8 ± 4.4	0.0	0.0	0.0	0.0	0.0	89.7
	-0.91	37.5 ± 5.9	55.0 ± 4.7	0.0	0.0	0.1	0.0	0.1	92.7
	-0.98	38.2 ± 5.9	58.7 ± 1.8	0.0	0.0	0.1	0.0	0.0	97.2
	-1.03	37.1 ± 5.3	61.3 ± 4.1	0.1 ± 0.0	0.0	1.4	0.0	2.2	102.1
**$Cu_{0.14}Zn_{0.86}O$**	-0.76	51.3 ± 5.0	37.2 ± 7.5	0.0	0.0	1.0	0.0	2.5	92.0
	-0.84	48.8 ± 3.6	38.2 ± 3.9	0.0	0.0	1.9	0.0	3.1	92.0
	-0.91	51.2 ± 5.4	43.9 ± 2.7	0.0	0.0	0.0	0.0	0.0	95.1
	-0.98	49.1 ± 4.2	45.5 ± 2.2	0.0	0.1 ± 0.1	0.1	0.0	1.9	96.7
	-1.03	50.2 ± 3.8	48.5 ± 3.2	0.3 ± 0.3	0.3 ± 0.3	0.8	0.0	0.8	100.9
**$Cu_{0.43}Zn_{0.57}O$**	-0.74	64.2 ± 3.0	28.6 ± 3.9	0.0	0.2 ± 0.2	0.9	0.2	0.0	94.0
	-0.81	59.7 ± 3.5	35.4 ± 2.2	0.0	0.1 ± 0.0	1.0	0.0	0.0	96.3
	-0.88	55.6 ± 3.0	42.2 ± 2.5	0.0	0.2 ± 0.1	0.7	0.2	0.3	99.2
	-0.93	54.7 ± 5.3	45.4 ± 4.1	0.1 ± 0.1	0.4 ± 0.1	1.0	0.0	0.9	102.5
	-0.98	56.1 ± 6.6	43.8 ± 7.4	0.4 ± 0.4	1.0 ± 0.4	1.7	0.1	1.0	104.2
**$Cu_{0.75}Zn_{0.25}O$**	-0.72	93.8 ± 2.3	6.3 ± 2.0	0.0	0.1 ± 0.1	0.0	2.6	0.6	103.6

	-0.78	91.6 ± 4.0	8.1 ± 2.6	0.0	0.1 ± 0.1	4.4	0.0	0.3	104.4
	-0.85	88.3 ± 2.6	12.6 ± 2.5	0.0	0.4 ± 0.3	0.8	1.0	0.6	103.7
	-0.92	82.3 ± 1.0	15.3 ± 2.5	0.5 ± 0.4	1.4 ± 0.7	0.6	2.4	1.0	103.6
	-0.96	75.0 ± 8.9	14.3 ± 2.8	4.7 ± 4.7	4.1 ± 1.7	0.8	0.3	2.7	101.9
**CuO**	-0.73	92.4 ± 3.7	2.1 ± 0.9	0.0	1.5 ± 0.7	4.0 ± 1.7	2.9 ± 2.1	1.6 ± 2.1	103.6
	-0.82	85.2 ± 5.6	2.7 ± 0.9	0.0	1.6 ± 0.2	3.5 ± 2.4	0.0	0.5 ± 0.6	93.1
	-0.91	73.0 ± 8.4	4.4 ± 1.1	0.4 ± 0.5	5.7 ± 0.9	3.6 ± 4.0	1.4 ± 1.1	1.5 ± 1.5	89.2
	-0.98	57.6 ± 9.4	5.6 ± 1.3	2.9 ± 2.7	13.6 ± 1.7	2.0 ± 2.4	3.4 ± 2.6	1.3 ± 0.9	84.7
	-1.04	42.4 ± 7.0	4.8 ± 1.4	9.1 ± 7.2	22.0 ± 2.5	6.4 ± 5.2	8.4 ± 4.4	5.7 ± 5.5	102.5

[1] Includes other CO_2RR products like ethane, methanol, acetate and 1-propanol.

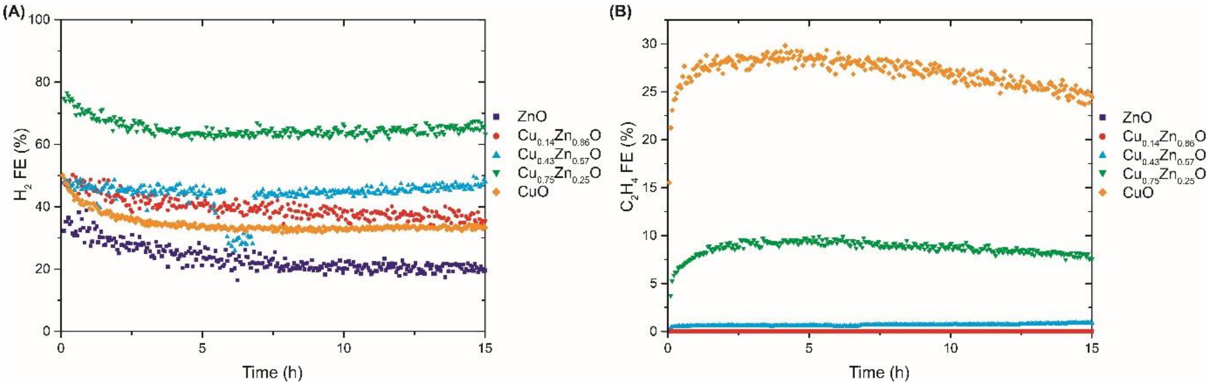

Figure S9. Faradaic Efficiency of H_2 (**A**) and C_2H_4 (**B**) over time during stability tests of the different catalysts for 15 hours.

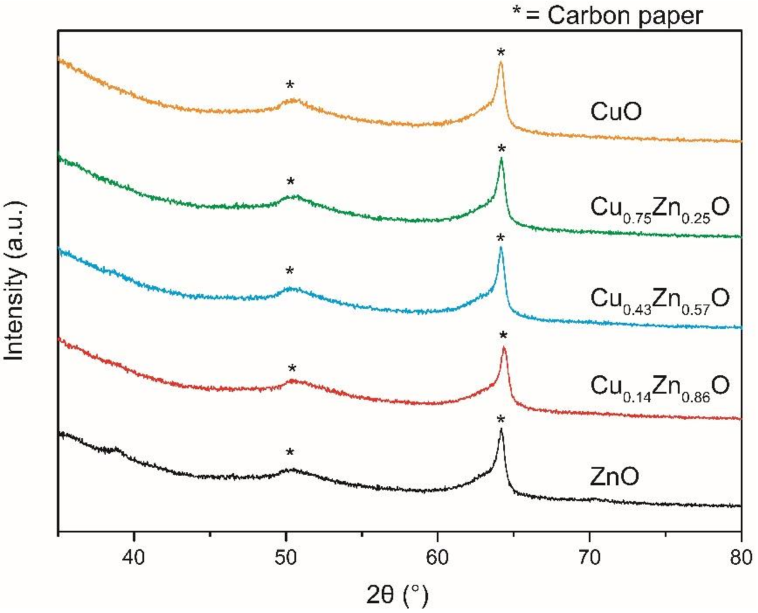

Figure S10. XRD patterns of the $Cu_{1-x}Zn_xO$ catalysts after catalysis. No copper- or zinc-related peaks are observed.

**Sample**	**Cu:Zn atom%**		**Metal loading on C paper (wt.%)**	
	**Fresh**	**Spent**	**Fresh**	**Spent**
ZnO	0	0	2.9	2.1
$Cu_{0.14}Zn_{0.86}O$	14	11	1.8	1.7
$Cu_{0.43}Zn_{0.57}O$	43	39	1.5	1.4
$Cu_{0.75}Zn_{0.25}O$	75	75	1.1	0.8
CuO	100	100	0.8	0.4

Table S3. ICP results of the electrodes before and after catalytic testing. No significant change in Cu:Zn ratio is observed, whereas some metal loss has taken place.

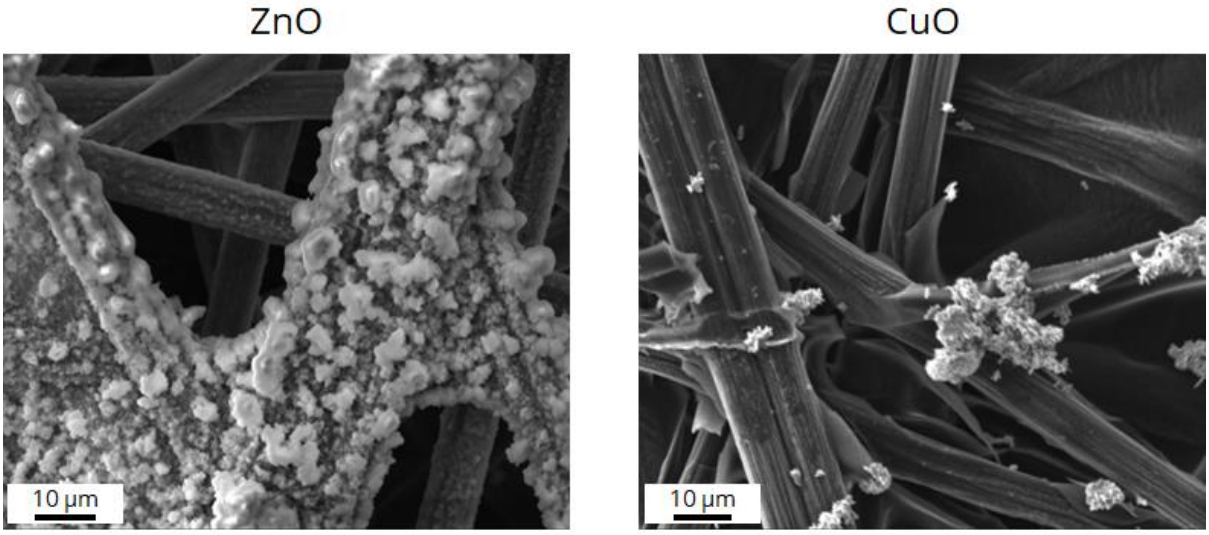

Figure S11. SEM images of ZnO and CuO electrodes after catalytic testing. Significant restructuring into amorphous structures has taken place compared to the pristine catalysts.

ZnO fresh

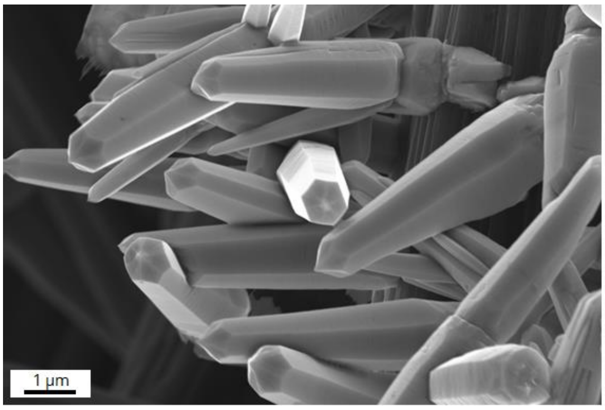

ZnO spent

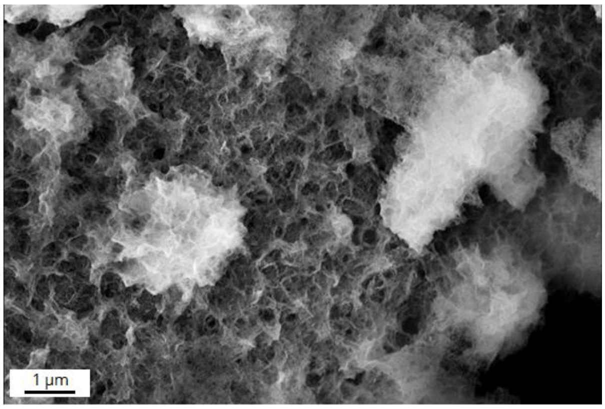

$Cu_{0.14}Zn_{0.86}O$ fresh

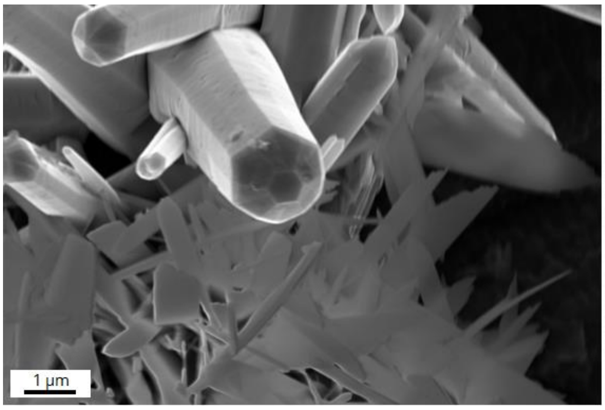

$Cu_{0.14}Zn_{0.86}O$ spent

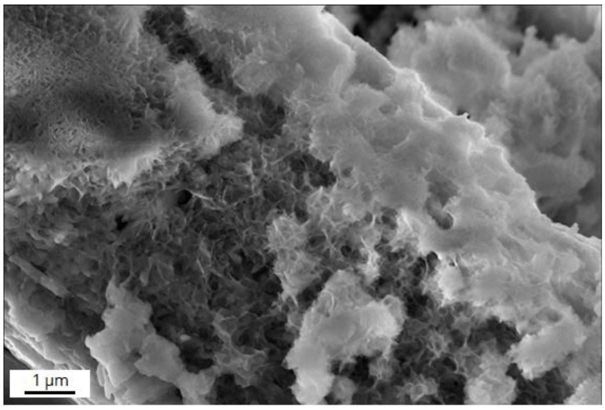

$Cu_{0.43}Zn_{0.57}O$ fresh

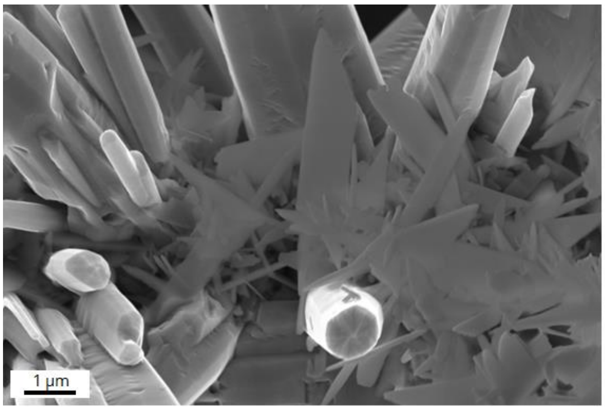

$Cu_{0.43}Zn_{0.57}O$ spent

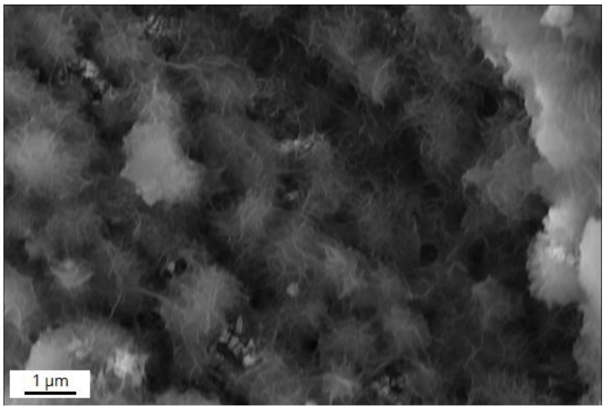

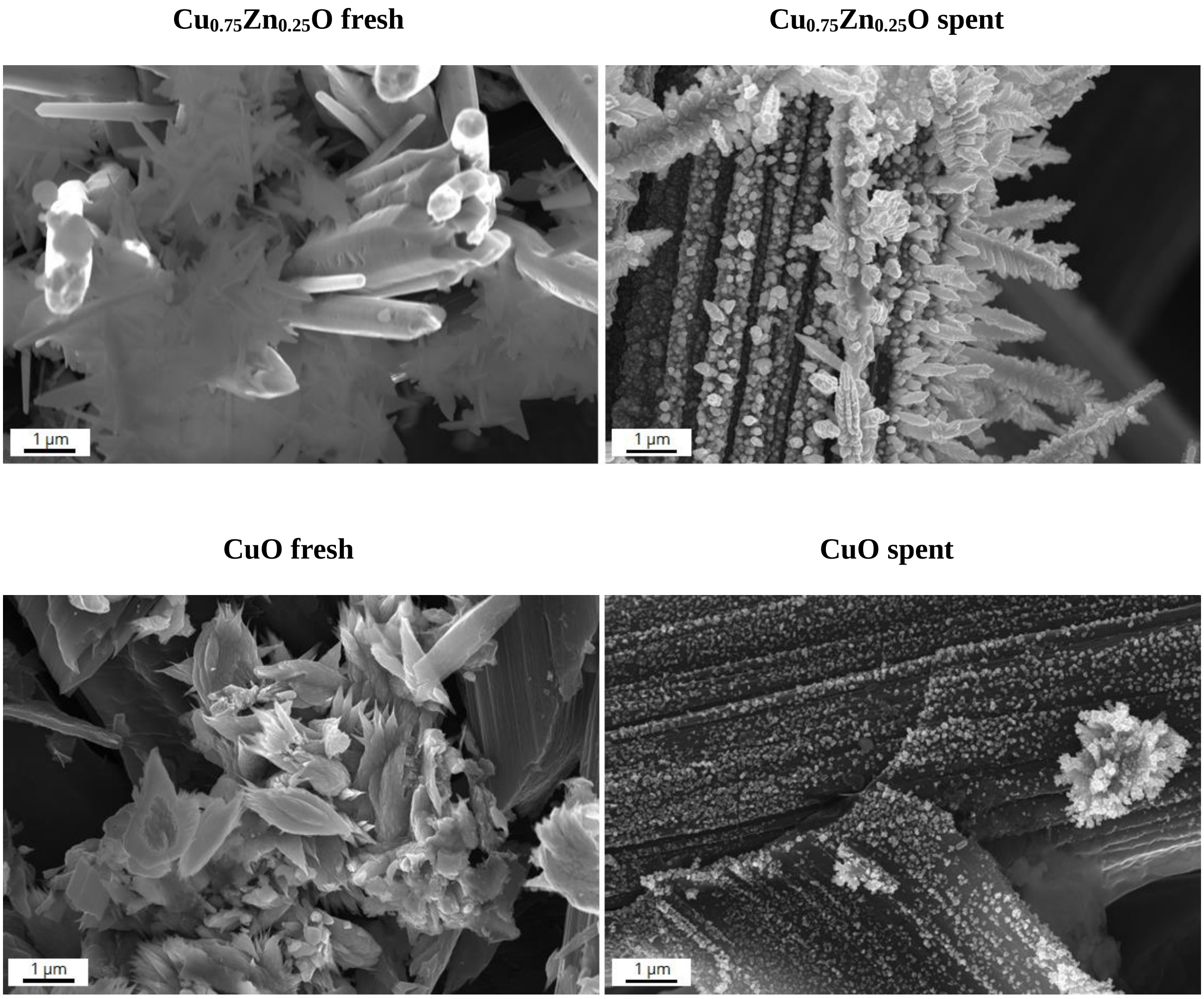

Figure S12. High-resolution SEM images of $Cu_{1-x}Zn_xO$ electrodes before (left) and after (right) catalytic testing.

ZnO

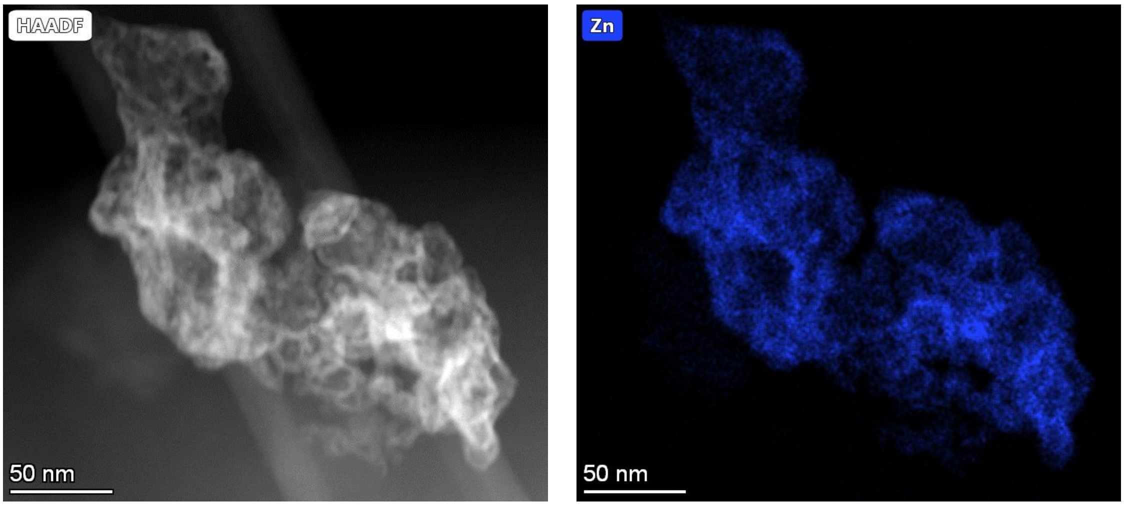

$Cu_{0.14}Zn_{0.86}O$

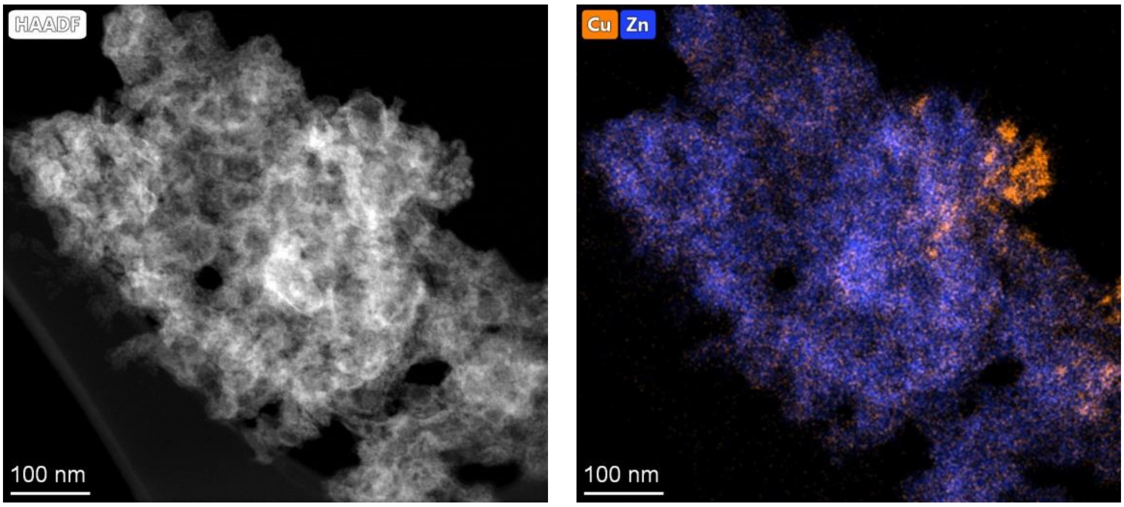

$Cu_{0.43}Zn_{0.57}O$

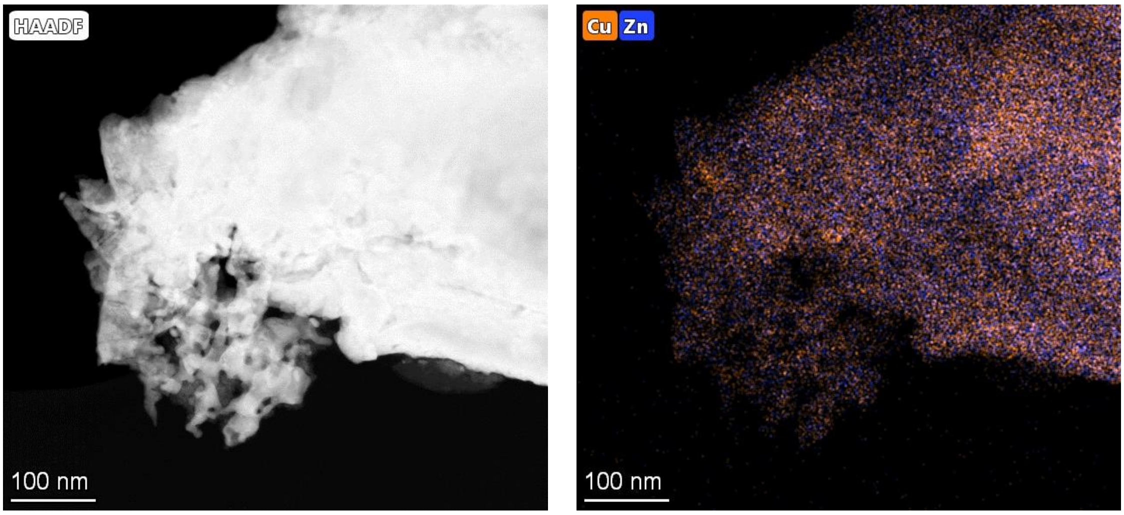

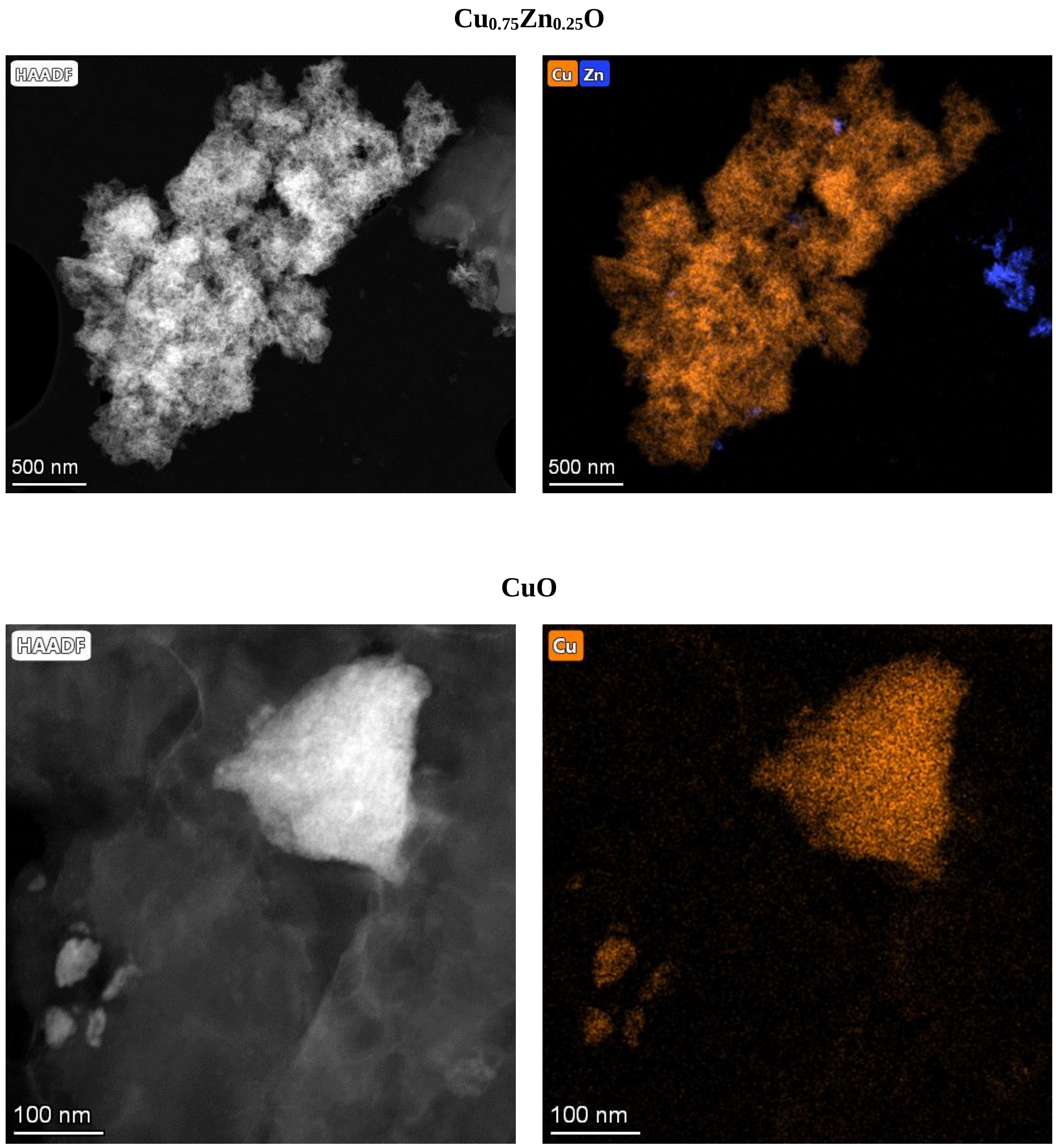

Figure S13. HAADF-STEM images (left) and corresponding EDX maps (right) of $Cu_{1-x}Zn_xO$ electrodes after catalytic testing, showing the element distributions of copper (orange) and zinc (blue).

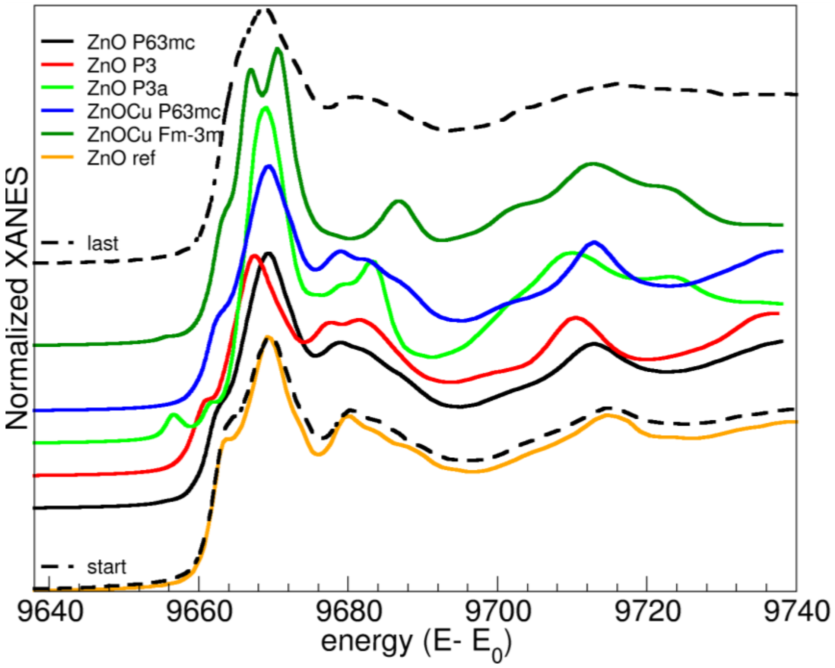

Figure S14. XANES Zn K-edge spectra of different Zn-O and ZnCu-O oxides. The solid lines in the figure point out the main contribution due to the cubic structure present in the experimental spectrum corresponding to the last electrochemical treatment of the $Cu_{0.14}Zn_{0.86}O$ catalyst at -0.6 V.

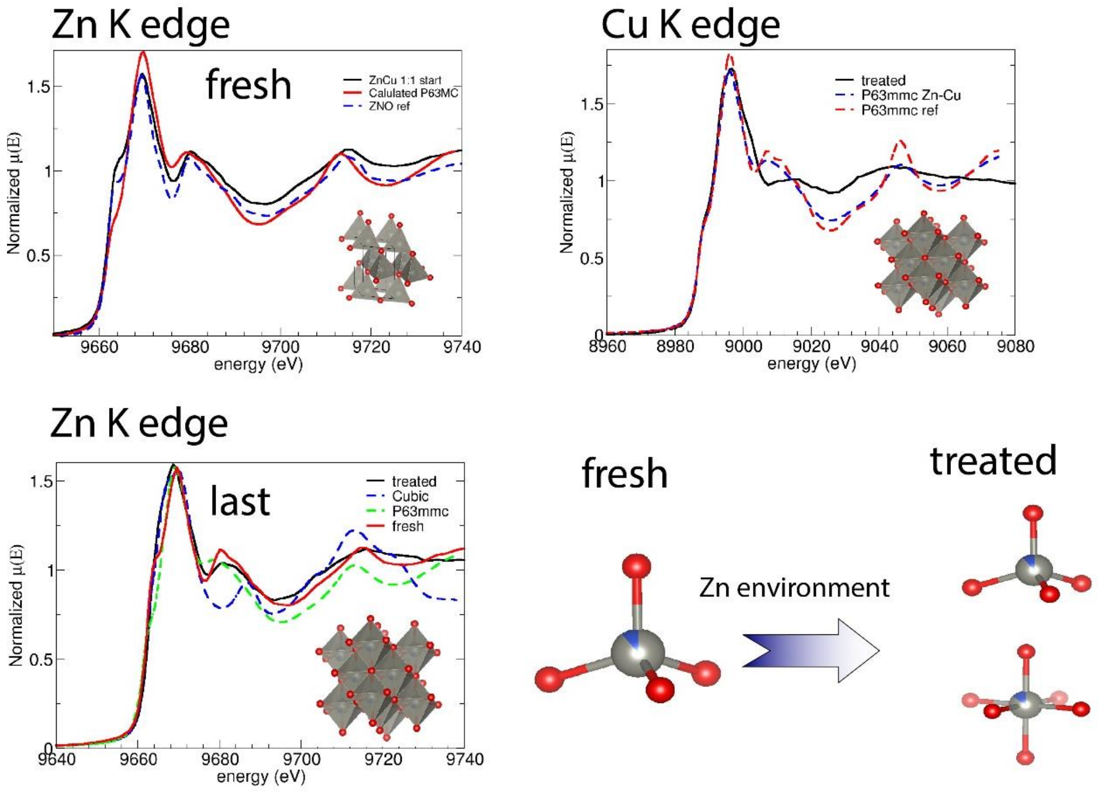

Fig S15. XANES Simulation of the Zn and Cu K edge of the fresh and treated $Cu_{0.14}Zn_{0.86}O$ catalyst, showing that zinc undergoes a structural transformation from tetrahedral to both tetrahedral and octahedral.

Table S4. EXAFS fitting results of Cu K-edge for $Cu_{0.14}Zn_{0.86}O$ catalyst under CO_2RR conditions.

**Potentials (V vs RHE)**	CN1	R1 (Å)	CN2	R2 (Å)	D cluster (nm)
**Pristine**	4.00	1.99	6.00	3.02	-
**0.4 V**	4.00	1.99	6.00	3.14	-
**0.2 V**	3.73	2.01	3.94	3.16	1.20
**0.1 V**	1.23	2.03	-	-	21.00
**0 V**	0.78	2.10	-	-	23.94
**-0.1 V**	0.56	2.14	-	-	23.42
**-0.4 V**	0.44	2.14	-	-	25.95
**-0.6 V**	0.95	2.17	-	-	2.00

Table S5. EXAFS fitting results of Zn K-edge for $Cu_{0.14}Zn_{0.86}O$ catalyst under CO_2RR conditions.

**Potentials (V vs RHE)**	CN1	R1 (Å)	CN2	R2 (Å)
**Pristine**	3.50	1.91	6.00	3.22
**0.4 V**	3.55	1.93	5.99	3.23
**0.2 V**	3.14	1.93	1.96	3.20
**0.1 V**	4.00	1.93	6.00	3.23
**0 V**	3.52	1.93	5.78	3.23
**-0.1 V**	3.56	1.93	6.00	3.22
**-0.4 V**	3.55	1.93	6.00	3.21
**-0.6 V**	3.43	1.98	0.25	3.14

CN is the coordination number of the reported scattering contribution.

R1 and R2 are the respective first-shell M-O, and second-shell M-O-M bond lengths.

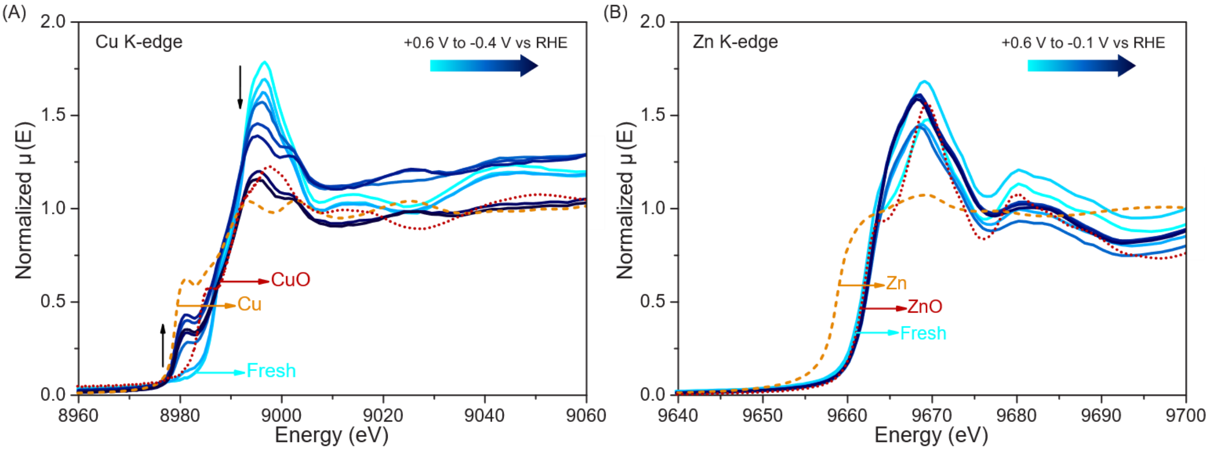

Figure S16. Potential dependence of operando (a) Cu K-edge and (a) Zn K-edge XANES spectra of $Cu_{0.43}Zn_{0.57}O$ and references in CO_2 saturated 0.1 M $KHCO_3$ under CO_2RR conditions

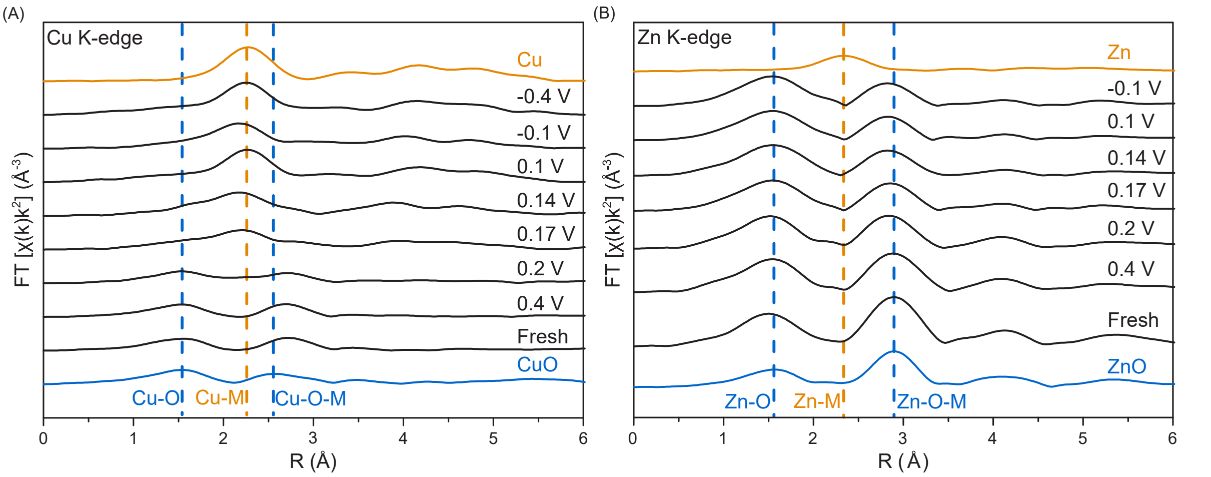

Figure S17. Potential dependence of Fourier-transformed (FT) EXAFS data at (a) Cu K-edge and (b) Zn K-edge for $Cu_{0.43}Zn_{0.57}O$ catalyst under CO_2RR conditions with increasingly cathodic potentials (V vs RHE). Data are not phase corrected.

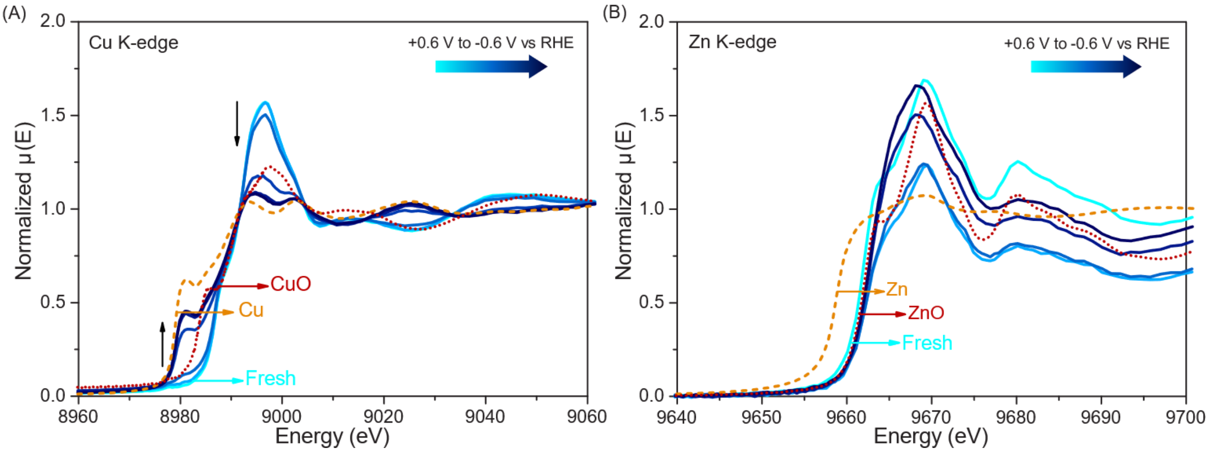

Figure S18. Potential dependence of *in-situ* (a) Cu K-edge and (a) Zn K-edge normalized XANES spectra of $Cu_{0.75}Zn_{0.25}O$ and reference materials in CO_2 saturated 0.1 M $KHCO_3$ under CO_2RR conditions

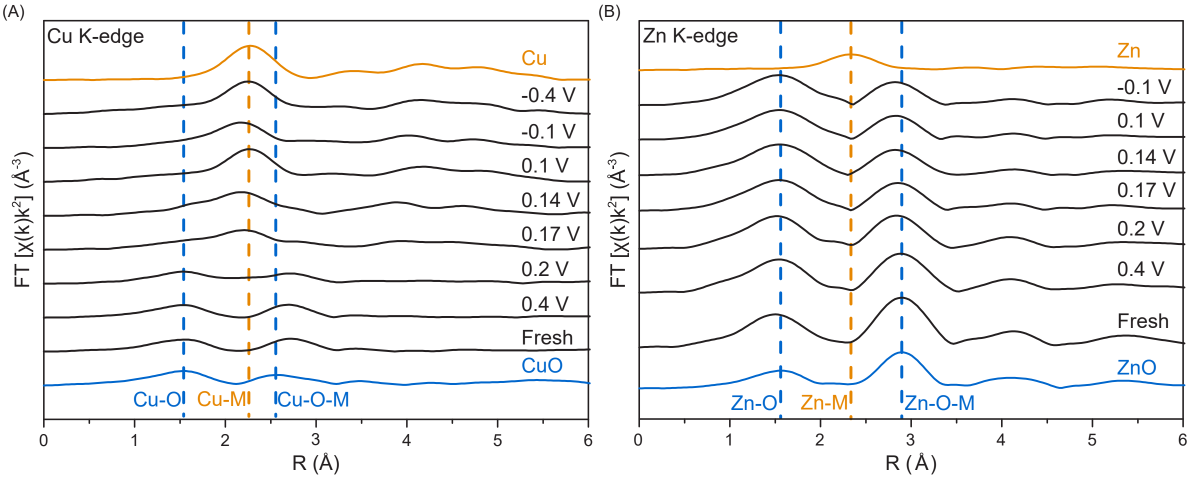

Figure S19. Potential dependence of Fourier-transformed (FT) EXAFS data at (a) Cu K-edge and (b) Zn K-edge for $Cu_{0.75}Zn_{0.25}O$ catalyst under CO_2RR conditions with increasingly cathodic potentials (V vs RHE). Data are not phase corrected.
